# Clusters of co-abundant proteins in the brain cortex associated with fronto-temporal lobar degeneration

**DOI:** 10.1186/s13195-023-01200-1

**Published:** 2023-03-23

**Authors:** Claire Bridel, Juami H. M. van Gils, Suzanne S. M. Miedema, Jeroen J. M. Hoozemans, Yolande A. L. Pijnenburg, August B. Smit, Annemieke J. M. Rozemuller, Sanne Abeln, Charlotte E. Teunissen

**Affiliations:** 1 Neurochemistry Laboratory and Biobank, Department of Clinical Chemistry, Amsterdam Neuroscience, Neurodegeneration, Amsterdam UMC, Amsterdam, The Netherlands; 2 Department of Clinical Neurosciences, Division of Neurology, Geneva University Hospital, Geneva, Switzerland; 3 Department of Computer Science, Bioinformatics group, VU University, Amsterdam, The Netherlands; 4 Department of Molecular and Cellular Neurobiology, Center for Neurogenomics and Cognitive Research, Neuroscience Campus Amsterdam, VU University, Amsterdam, The Netherlands; 5 Department of Pathology, Amsterdam Neuroscience, Amsterdam UMC, Amsterdam, The Netherlands; 6 Alzheimer Center, Department of Neurology, Amsterdam Neuroscience, Amsterdam UMC, Amsterdam, The Netherlands

**Keywords:** Frontotemporal lobar degeneration, TDP43, Tau, Dementia, Tissue proteomics, Co-expression module, Chromatin regulation, Clathrin-mediated transport, PTBP1, CDK5

## Abstract

**Background:**

Frontotemporal lobar degeneration (FTLD) is characterized pathologically by neuronal and glial inclusions of hyperphosphorylated tau or by neuronal cytoplasmic inclusions of TDP43. This study aimed at deciphering the molecular mechanisms leading to these distinct pathological subtypes.

**Methods:**

To this end, we performed an unbiased mass spectrometry-based proteomic and systems-level analysis of the middle frontal gyrus cortices of FTLD-tau (*n* = 6), FTLD-TDP (*n* = 15), and control patients (*n* = 5). We validated these results in an independent patient cohort (total *n* = 24).

**Results:**

The middle frontal gyrus cortex proteome was most significantly altered in FTLD-tau compared to controls (294 differentially expressed proteins at FDR = 0.05). The proteomic modifications in FTLD-TDP were more heterogeneous (49 differentially expressed proteins at FDR = 0.1). Weighted co-expression network analysis revealed 17 modules of co-regulated proteins, 13 of which were dysregulated in FTLD-tau. These modules included proteins associated with oxidative phosphorylation, scavenger mechanisms, chromatin regulation, and clathrin-mediated transport in both the frontal and temporal cortex of FTLD-tau. The most strongly dysregulated subnetworks identified cyclin-dependent kinase 5 (CDK5) and polypyrimidine tract-binding protein 1 (PTBP1) as key players in the disease process. Dysregulation of 9 of these modules was confirmed in independent validation data sets of FLTD-tau and control temporal and frontal cortex (total *n* = 24). Dysregulated modules were primarily associated with changes in astrocyte and endothelial cell protein abundance levels, indicating pathological changes in FTD are not limited to neurons.

**Conclusions:**

Using this innovative workflow and zooming in on the most strongly dysregulated proteins of the identified modules, we were able to identify disease-associated mechanisms in FTLD-tau with high potential as biomarkers and/or therapeutic targets.

**Supplementary Information:**

The online version contains supplementary material available at 10.1186/s13195-023-01200-1.

## Background

Frontotemporal dementia (FTD) is a pathologically heterogeneous disease, clinically characterized by progressive behavioural and/or language alterations with relative memory sparing [1]. Pathologically, neurodegeneration predominates in the frontal and temporal lobes (frontotemporal lobar degeneration (FTLD)) and correlates with aberrant protein aggregation and cellular inclusion formation [2]. FTLD is mostly sporadic, but a positive family history (familial FTLD (fFTLD)) is identified in 25–40%, with an autosomal dominant pattern of inheritance in 10–15% [1]. Two major non-overlapping pathological subtypes of FTLD have been identified regardless of the sporadic or familial nature of the disease: FTLD-tau, characterized by neuronal and glial inclusions of hyperphosphorylated tau, and FTLD-TDP, characterized by neuronal inclusions of hyperphosphorylated transactive response DNA-binding protein 43 kDa (TDP43) [2]. The underlying pathology cannot be anticipated from the clinical phenotype in sporadic FTLD, but in autosomal dominant fFTLD, it can be inferred from the genotype. Mutations in the chromosome 9 open reading frame gene (*C9ORF*), the progranulin gene (*GRN*), and the TDP43 gene (*TARDB*) invariably lead to FTLD-TDP pathology, while mutations in the microtubule-associated protein tau gene (*MAPT*) consistently lead to an FTLD-tau pathological signature [1]. The heterogeneity of these genetic features and the pathological similarity between sporadic and (autosomal dominant) fFTLD indicate different initiating events can converge towards a comparable pathological end-point characterized either by tau or TDP43 aggregates.

Immunohistochemistry reveals that the types of cytoplasmic inclusions within the frontal and temporal lobes observed in FTLD-TDP can roughly be divided into four subcategories (A–D) [3]. Interestingly, recent studies have indicated that cerebellar TDP-43 is also observed in FTD [4, 5]. While progress has been made in the pathological characterization of FTLD, there is still limited insight into the molecular mechanisms leading to tau or TDP-43 pathology in the FTLD brain [6]. One of these mechanisms is the disruption of nuclear pore complexes and nucleocytoplasmic transport by the formation of TDP-43 aggregates through sequestration or mislocalisation of factors important for nuclear transport or nuclear pore integrity [7]. Another disease mechanism is the incorrect splicing of RNA of other genes due to loss of function and consequent lower levels of mutated TDP-43 [8, 9].

Disease mechanisms for FTLD-tau may involve reduced interaction of tau with mitochondrial proteins [10], lysosomal dysfunction [11], and glutamatergic dysfunction [12].

Identifying the mechanisms and key molecules leading to neuronal death associated with tau or TDP43 aggregates is of foremost importance in the development of specific therapies for FTLD, for which there are currently none [13].

Proteomic approaches allow to investigate the global changes in protein abundance levels in human tissues, offering an attractive entry point into the discovery of molecular pathways associated with a pathological phenotype. Few studies have used systems-level proteomic analysis to identify the dysregulated molecular pathways associated with FTLD, and none so far has compared the two most prevalent pathological subtypes of FTLD [14]. To identify the dysregulated pathways and cell-type abundance changes associated with FTLD-tau and/or FTLD-TDP, we investigated a postmortem discovery cohort consisting of sporadic or autosomal dominant FTLD cases with tau (*n* = 6) or TDP (*n* = 15) underlying pathology and age-matched neurologically healthy controls (NHC, *n* = 5). The frozen middle frontal gyrus cortex, selected because it is consistently affected in FTLD, was microdissected and subjected to data-independent quantitative proteomics analysis. In addition to differential expression analysis and gene set enrichment analysis, we performed a systems-level analysis of the entire proteome. To this end, we developed an original workflow to identify co-regulated modules of proteins as well as the most strongly differentially expressed subnetworks of proteins within these modules. We designed a novel method to validate the protein modules in two data sets originating from the middle frontal gyrus cortex and temporal cortex samples of an independent validation cohort of individuals with FLTD-tau and NHC (*n* = 24) and identified the cell types most significantly associated with the dysregulated modules.

## Materials and methods

### Overview of the workflow

**Fig. 1:**
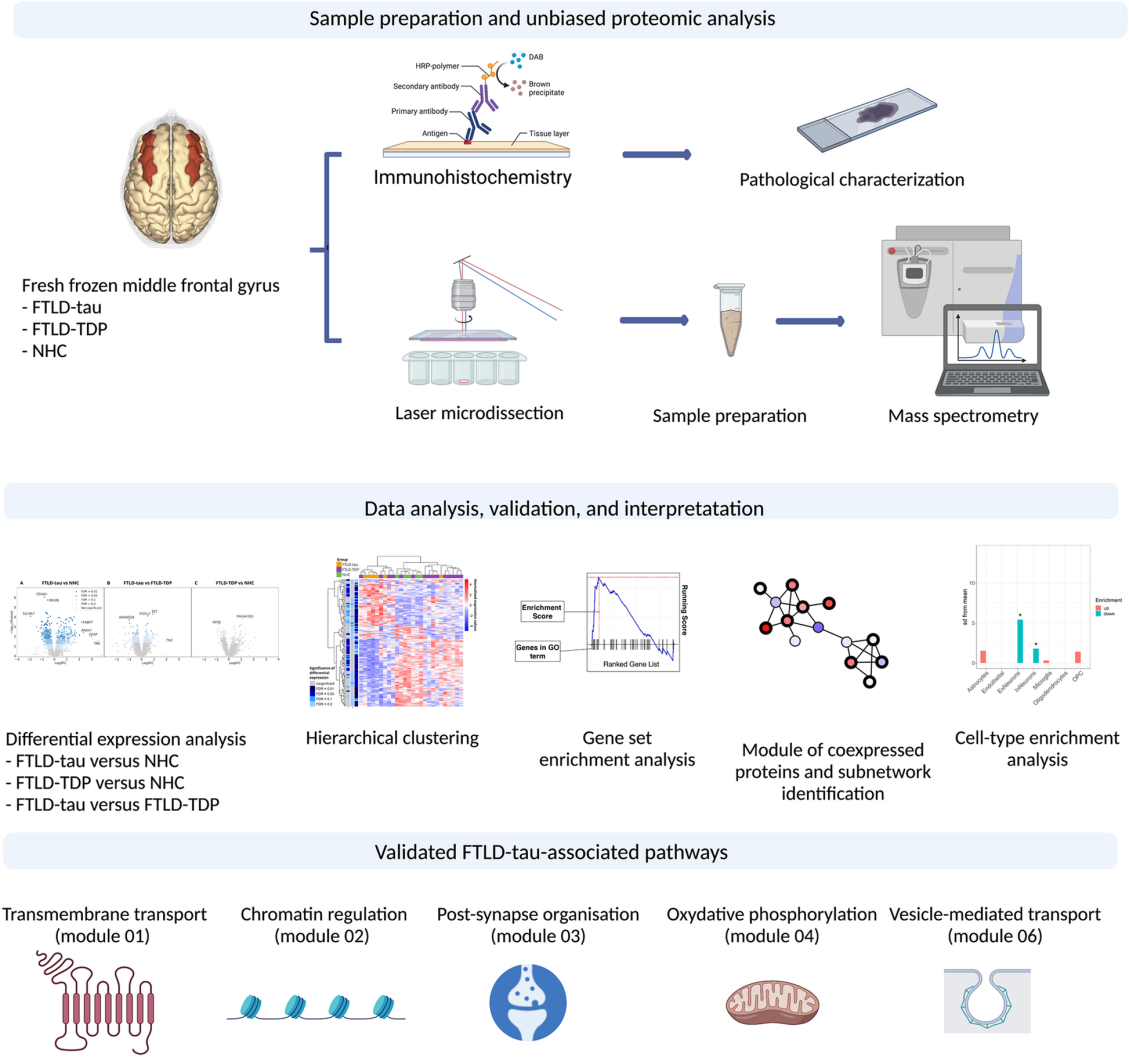
Overview of the workflow. Tissue sections of fresh frozen medial frontal gyrus were microdissected to isolate the cortex. Proteins were quantified using LC-MS/MS. Differentially expressed proteins were identified by comparing protein abundance levels across the 3 diagnostic groups. GSEA was performed to identify the classes of proteins enriched in FTLD-tau and/or FTLD-TDP. WGCNA was performed to identify the modules of co-regulated proteins. Based on these modules, cell-type enrichment analysis was performed to identify the cell type most significantly associated with the protein modules. Finally, modules were validated using 2 validation data sets (frontal validation data set and temporal validation data set)

An overview of the study workflow is provided in Fig. 1. In brief, a small section of the middle frontal gyrus cortex was microdissected from fresh frozen brain samples originating from the discovery cohort (Table S1). In parallel, immunohistochemistry was performed on consecutive sections to ensure the region analysed by mass spectrometry was affected with the characteristic neuropathological changes. Proteins were extracted from the samples and quantified using SWATH mass spectrometry. Computational analysis included differential expression analysis between the 3 diagnostic groups, gene set enrichment analysis (GSEA) [15] to identify classes of genes (proteins) overrepresented in FTLD-tau and/or FTLD-TDP, identification of co-regulated protein modules using weighted gene coexpression network analysis (WGCNA) [16], determination and validation of the most strongly differentially expressed subnetworks within each module, and expression weighted cell type enrichment (EWCE) [17] analysis to determine which central nervous system (CNS) cell types were most significantly associated with the dysregulated protein modules.

### Discovery cohort

Fresh frozen brain tissue of the middle frontal gyrus was obtained from the Netherlands Brain Bank, Netherlands Institute for Neuroscience, Amsterdam. All brain donors had given written informed consent for brain autopsy and the use of material and clinical information for research. The discovery cohort consisted of 26 cases which were selected based on detailed neuropathological postmortem assessment and clinical information. Pure FTLD-tau or FTLD-TDP without significant age-associated pathologies including Alzheimer’s disease pathology, cerebral amyloid angiopathy, Lewy body disease, and vascular pathology were selected. All FTLD-tau cases (*n* = 6) were carriers of a MAPT mutation, and of the 15 FTLD-TDP cases, 3 were sporadic, 2 were *GRN* mutation carriers, 8 were *C9ORF* mutation carriers, and 2 were familial (without *GRN*, *C9ORF*, or Valosin-containing protein (*VCP*) mutations). Age-matched NHC (*n* = 5) were selected among individuals who died without signs or symptoms of a neurological condition (Table S1), as defined by the Netherlands Brain Bank (https://www.brainbank.nl/brain-tissue/diagnostics/#item-2). The three pathological groups were matched in sex, age, and PMI (Table S3).

### Validation data sets

Two validation data sets were used. The first data set consisted of proteomic abundance data of the microdissected middle frontal gyrus cortex originating from 11 autosomal dominant fFTD with underlying FTLD-tau pathology and 11 NHC individuals (Table S1). The second data set consisted of proteomic abundance data of the microdissected temporal cortex originating from 13 autosomal dominant fFTD individuals with FTLD-tau underlying pathology and 8 NHC (Table S1). These data sets were selected from a larger data set that was acquired following the same brain sample preparation protocol, protein-to-peptide procedure, proteomic workflow, and instruments as in this study, but analysed in a different batch [18]. Access to the full data set of the validation cohort is provided in the original publication [18]. Note that the validation sets are partially paired, where from 2 subjects, only the temporal cortex was extracted; from 3 samples, only the frontal cortex was extracted; and from the remaining 19, both the frontal and temporal cortices were extracted (Table S1).

### Immunohistochemistry

Fresh frozen human postmortem middle frontal gyrus was cut (5 μm sections), placed on a StarFrost Microscope Slide (Knittel Glass), and air dried overnight at room temperature. Before staining, the sections were fixed in 100% acetone for 10 min. Endogenous peroxidase activity was quenched by incubation with 0.3% (vol/vol) H_2_O_2_in methanol for 30 min at room temperature. Antigen retrieval was performed by soaking the sections in 10 mM citrate buffer pH 6.0, heated, and boiled for 10 min in a microwave oven. The sections were stained overnight at 4 °C with primary antibodies against amyloid beta, phosphorylated tau, p62, and TDP43, as described previously [19]. Primary antibodies were visualized using Power vision (Dako, Denmark).

### Preparation of brain homogenates

Ten-micrometre sections of fresh frozen tissue consecutive to those used for immunohistochemistry were mounted on polyethylene naphthalate-membrane slides (Leica, Herborn, DE), fixed in 100% ethanol for 1 min, and stained using 1% (wt/vol) Toluidine Blue in H_2_O (Fluka Analytical, Buchs, Switzerland) for 1 min, according to an in-house developed protocol [20]. The middle frontal gyrus cortex (50 mm^3^) was isolated from white matter using laser micro-dissection with a Leica AS LMD system. The sections were collected in Eppendorf tubes containing 30 mL M-PER lysis buffer (Thermo Scientific, Rockford, IL, USA) supplemented with reducing sodium dodecyl sulphate sample buffer (Thermo Scientific). Samples were boiled at 95 °C for 5 min and then centrifuged. The supernatant was stored at − 80 °C until further use. Protein separation by electrophoresis, in‑gel digestion, and peptide extraction were done as described previously [21]. Samples were diluted such that the total amount of protein in each sample used for mass spectrometry was 20 μg.

### Liquid chromatography tandem mass spectrometry

Peptides were analysed by liquid chromatography tandem mass spectrometry (LC-MS/MS) using an Ultimate 3000 LC system (Dionex, Thermo Scientific) coupled to the Triple TOF 5600 mass spectrometer (Sciex). Peptides were trapped on a 5-mm Pepmap 100 C18 column (300 μm i.d., 5 μm particle size, Dionex) and fractionated on a 200-mm Alltima C18 column (300 μm i.d., 3 μm particle size). The acetonitrile concentration in the mobile phase was increased from 5 to 18% in 88 min, 25% at 98 min, 40% at 108 min, and 90% in 2 min, at a flow rate of 5 μL/min. The eluted peptides were electro-sprayed into the Triple TOF MS. The micro-spray needle voltage was set to 5500 V. SWATH experiments consisted of a parent ion scan of 150 ms followed by a SWATH window of 8 Da with a scan time of 80 ms and stepped through the mass range between 450 and 770 m/z. The total cycle time was about 3.2 s, which yielded in general 9–10 measurement points across a typical peptide with an elution time of 30 s. The collision energy for each window was determined based on the appropriate collision energy for a 2+ ion, centred upon the window with a spread of 15 eV.

### Proteomic data analysis

Spectronaut 11 was used to process the SWATH data [22]. Apart from the parameters explicitly mentioned here, Spectronaut was run using the default settings. The retention time prediction type was set to dynamic iRT, and interference correction was enabled. Finally, across-run normalization based on peptide total peak areas was performed by Spectronaut. Peptide abundances and *Q*-values (quality score, SWATH peptide to spectral library matching) were exported as a Spectronaut report and further processed using the R language for statistical computation. Within each fraction, peptides with *Q*-value > 0.01 in more than half the samples were discarded to assure high-confidence annotations of the MS signals to the peptides. Quality control of the peptides for a few selected proteins is shown in Table S3. Next, fractions were combined by summation of same-peptide abundance, and subsequently, protein-level abundances were computed by summation of their top 5 (overall) most abundant peptides. The protein abundances were normalized such that the total protein count was equal for all the samples. Both because the total amount of protein injected into the system (see the “Preparation of brain homogenates” section) and because LC-MS/MS typically measures the relative protein abundance rather than the absolute values [23], this normalization is necessary to avoid biases in downstream analyses.

### Hierarchical clustering

Protein abundances were first normalized for total protein count per sample then centred and scaled. Pearson correlations between both the samples and the proteins were calculated and used as a distance measure for clustering. Hierarchical clustering was performed using the “hclust” function with average linkage in R version 3.6.1.

### Differential expression analysis

Differential expression analysis was performed in pairwise comparisons using the beta-binomial test (ibb R package version 13.6) [24]. A beta-uniform mixture model was fitted to the *p*-value distribution of each of the comparisons to determine the FDR (BioNet R package version 1.40.0).

### Gene set enrichment analysis

GSEA was performed using the GSEA software version 4.0.3 “Run GSEA Preranked” tool with 1000 permutations, on the preranked log2 fold change (log2FC) protein levels resulting from the three pairwise comparisons (FTLD-tau versus NHC, FTLD-TDP versus NHC, and FTLD-tau versus FTLD-TDP). Normalized enrichment scores (NES) were calculated as described [15]. To gain complementary information, 5 different gene set databases from the GSEA Molecular Signatures Database (msigdb version 6.2) were used: the Chemical and Genetic Perturbations (CGP) database, which consists of gene sets functionally related to phenotypes or diseases; the Kyoto Encyclopedia of Genes and Genomes (KEGG) and the REACTOME databases, which list, in addition, gene sets associated with metabolic pathways; and the Gene Ontology (GO) biological process (GO-bp) and molecular function (GO-mf) databases. The CGP Alzheimer’s disease gene set “blalock alzheimers disease up” results from a microarray analysis of the human postmortem hippocampus of Alzheimer’s disease individuals and controls and includes 1668 genes upregulated in Alzheimer’s disease compared to controls [25]. The CGP ageing frontal cortex data set “lu ageing brain up” results from a microarray analysis of the human postmortem frontal cortex of individuals ranging from 26 to 106 years of age and includes 261 genes upregulated in the ageing frontal cortex [26]. The CGP Huntington’s disease data set (*n* = 15 genes) and KEGG Huntington’s disease data set (*n* = 182 genes) include genes involved in clathrin-mediated endocytosis and the endo-lysosomal and proteasome pathway and neurotransmitter signalling. The KEGG Parkinson’s disease data set (*n* = 130 genes) and CGP Parkinson’s disease data set (*n* = 84 genes) include genes mutated in familial Parkinson’s disease and genes belonging to the molecular pathways regulated by these genes.

### Identification of co-regulated protein modules

A limitation of GSEA is that it requires pre-annotated functional groups of genes, which biases the analysis towards known pathways and limits the ability to identify new functional groups of genes. To overcome this limitation and identify the groups of co-regulated proteins in an unsupervised manner without requiring predefined functional groups or sample labels, we designed a novel workflow combing WGCNA and Hierarchical HotNet [16, 27]. We used the WGCNA R package version 3.6.1 to create coexpression networks based on the sample-normalized protein abundance levels (Fig. S1, top row). A similarity matrix of the protein abundance levels was created by calculating the absolute value of the Pearson correlations between protein abundance levels and taking these values to the 7th power (this power was determined using the “pickSoftThreshold” function in the package). Based on the similarity matrix, a hierarchical tree of the proteins was created. Subsequently, the dynamic tree cut method was used to divide the proteins into modules of co-regulated proteins. After the modules were created, GO enrichment analysis was performed on each module to identify functional enrichment. This way, modules can be created in an unbiased fashion while still gaining functional insight into the module content. All proteins in our data set with at least one associated GO term were used as background for the enrichment test. To determine whether a module was significantly differently abundant between the diagnostic groups, we performed Student’s *t*-test with Benjamini-Hochberg correction on the distribution of the log2FC values of all proteins within that module (Fig. S1). We define a module to be dysregulated if this distribution is significantly different from zero (i.e. there is a consistent trend of the module proteins to have higher abundance in one pathological group compared to another).

#### Determination of differentially abundant subnetworks

Most of the modules of co-regulated proteins consisted of > 100 proteins and were thus too large to be interpreted easily. Furthermore, many proteins in each module had only low to medium absolute log2FC values. We developed a method to overcome these limitations and identify subnetworks of proteins most significantly dysregulated and thus more likely to have a high functional influence in the module from which they originate. Using correlations between protein abundance levels within each module as edges, a network of all the proteins within a module was created. Using Hierarchical HotNet, we identified subnetworks of proteins most highly and significantly dysregulated in each module based on the *p*-value between FTLD-tau and NHC for each of the proteins (Fig. S1) [16]. Subnetworks were visualized using Cytoscape. Note that because the number of proteins in each subnetwork consists of a fraction of the number of proteins within the modules from which it originates, the proteins within the subnetwork do not necessarily reflect the overall GO term(s) associated with the module. The number of proteins in the subnetwork that have a GO annotation that supports the module GO term is shown in Table S2. Separate GO term enrichment analysis on the subnetworks was not performed because the subnetworks are generally too small to perform a meaningful enrichment analysis.

#### Module validation

The differential abundance of the modules was validated in the frontal and temporal validation data sets (Table S2). Data pre-processing and differential expression analysis were performed in the same way as for the discovery data set. The log2FC and *p*-values obtained for the contrasts between FTLD-tau and NHC in both validation data sets were mapped onto the modules identified in the discovery data set. We then counted the fraction of proteins (${F}_{m,t}$) within module $m$ that had a *p*-value lower than a given threshold $t$ and a differential expression level expressed in the same direction (negative or positive log2FC value) in both data sets (Fig. S2):

$${F}_{m,t}=\frac{{\sum }_{P\in m}1 if \left({p}_{P,d}<t \wedge {p}_{P,v}<t \wedge {FC}_{P,d}\bullet {FC}_{P,v}>0 \right)}{{N}_{m}}$$

where *P* $\in$ m indicates the set of proteins in module $m$, ${p}_{P,d}$ is the *p*-value of protein $\mathrm{P}$ in the discovery data set, ${p}_{P,v}$ is the *p*-value of protein P in the validation data set, ${{log}_{2}FC}_{P,d}$ is the log2 fold change of protein P in the discovery data set, ${{log}_{2}FC}_{P,v}$ is the log2 fold change of protein $P$ in the validation data set, and ${N}_{m}$ is the total number of proteins in module $m$.

Subsequently, we randomly shuffled the protein labels and calculated ${F}_{m,t}$ for the shuffled data. We repeated this permutation 1000 times. Subsequently, we calculated whether ${F}_{m,t}$ was significantly higher in the real data set compared to the permuted data at *p*-value thresholds of 0.1, 0.2, 0.5, and 0.8 using a one-sided *t*-test. If the average of these four *p*-values, which we call the gene expression set similarity (GESS) score, is lower than 0.05, we considered the module validated (Fig. S2). Note that in the context of this work, validation refers to the validation of protein abundance patterns within each module, rather than the module or subnetwork topology. We were interested in validating the modules in terms of pathological differences in protein abundances, and linking those to biological function through GO term analysis, rather than the exact module/subnetwork topologies.

### Expression-weighted cell type enrichment analysis

To determine if any of the modules could be linked to changes in the relative abundance of proteins associated with specific cell types between pathological groups, EWCE analysis of differentially expressed proteins was performed based on a data set of single nuclei RNA sequencing (RNAseq) of adult NHC postmortem frontal cortex [28]. Pre-processing and analysis of small non-coding RNAseq (snRNAseq) data sets was performed using the Python package Scanpy version 1.5.1 as described previously [29, 30]. Cell-gene matrices were filtered for outliers, and gene expression was normalized per cell. All cells were clustered using Louvain clustering implementation on the top 1000 highly variable genes. To identify cell types, marker genes and expected cell types were inferred from the original publications of the data sets. Angiotensinogen (AGT), electrogenic sodium bicarbonate cotransporter 1 (SLC4A4), and excitatory amino acid transporter 2 (SLC1A2) were used as markers of astrocytes; vascular endothelial growth factor receptor 1 (FLT1), dual specificity protein phosphatase 1 (DUSP1), and nostrin (NOSTRIN) as markers of endothelial cells; vesicular glutamate transporter 1 (SLC17A7) as a marker of excitatory neurons; glutamate decarboxylase 1 (GAD1) as a marker of inhibitory neurons; amyloid beta A4 precursor protein-binding family B member 1-interacting protein (APBB1IP) and TYRO protein tyrosine kinase-binding protein (TYROBP) as markers of microglia; myelin-associated oligodendrocyte basic protein (MOBP) as a marker of oligodendrocytes; and protocadherin-15 (PCDH15) and platelet-derived growth factor receptor alpha (PDGFRA) as markers of oligodendrocyte precursor cells (OPCs). Clusters that could not be clearly identified with one cell type were labelled “unknown”. Normalized gene expression data and cell type label matrices were subsequently used for expression-weighted cell type enrichment analysis using the EWCE package in R version 3.14 [17]. The total set of SWATH quantified proteins for FTLD-tau and FTLD-TDP was used as the background set, from which 20,000 random lists were generated for bootstrapped analysis of the probability distribution of cell type abundance. The standard deviation of the average expression of proteins associated with a cell type compared to the mean expression of bootstrapped gene lists is used as a measure of significance of the cell type enrichment [17].

## Results

### Quantitative proteomics of the medial frontal gyrus cortex reveals the largest contrast between FTLD-tau and NHC

**Fig. 2:**
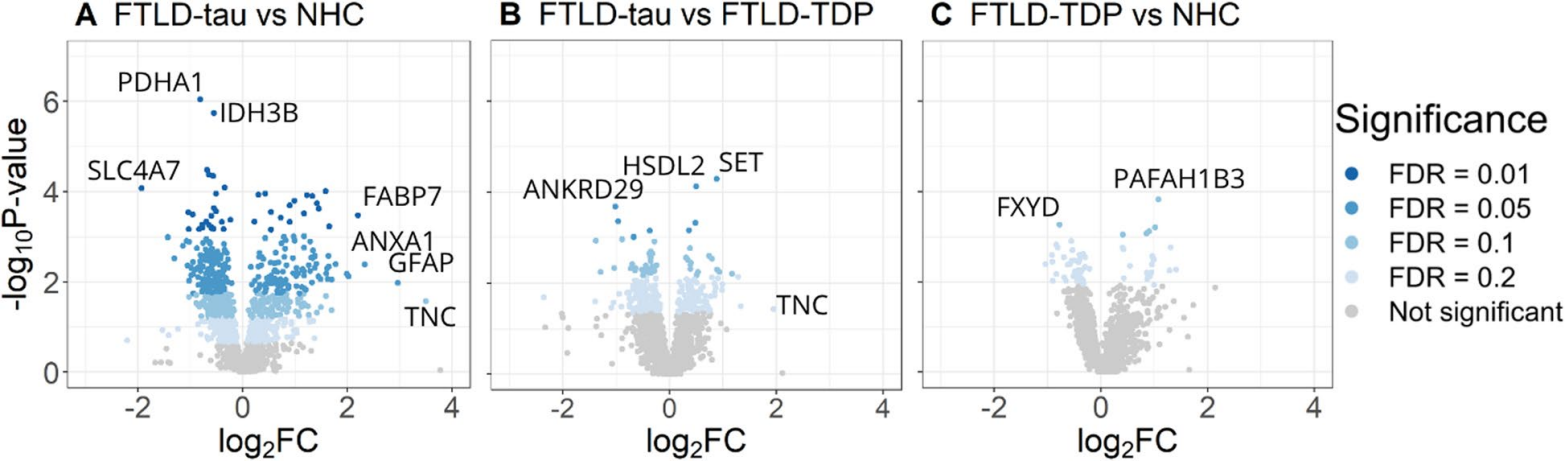
Volcano plot of pairwise proteome comparisons. Distribution of the log2 fold change (log2FC) protein abundance level (*x*-axis) and corresponding *p*-value (*y*-axis) for the proteome comparison between **A** FTLD-tau and NHC, **B** FTLD-tau and FTLD-TDP, and **C** FTLD-TDP and NHC. Levels of significance are indicated by the blue colour: the darker the colour, the more significant the contrast, with corresponding false discovery rates (FDR) indicated in the legend. The most highly and significantly dysregulated proteins are indicated by their names. SLC4A7, solute carrier family 4 member 7; FABP7, fatty acid-binding protein 7; ANXA1, Anexin 1; GFAP, glial fibrillary acid protein; TNC, Tenascin C; PDHA1, pyruvate dehydrogenase E1 subunit alpha 1; IDH3B, isocitrate dehydrogenase (NAD(+)) 3 non-catalytic subunit beta; ANKRD29, ankyrin repeat domain 29; HSDL2, hydroxysteroid dehydrogenase like 2; PAFAH1B3, platelet-activating factor acetylhydrolase IB subunit alpha1

**Fig. 3:**
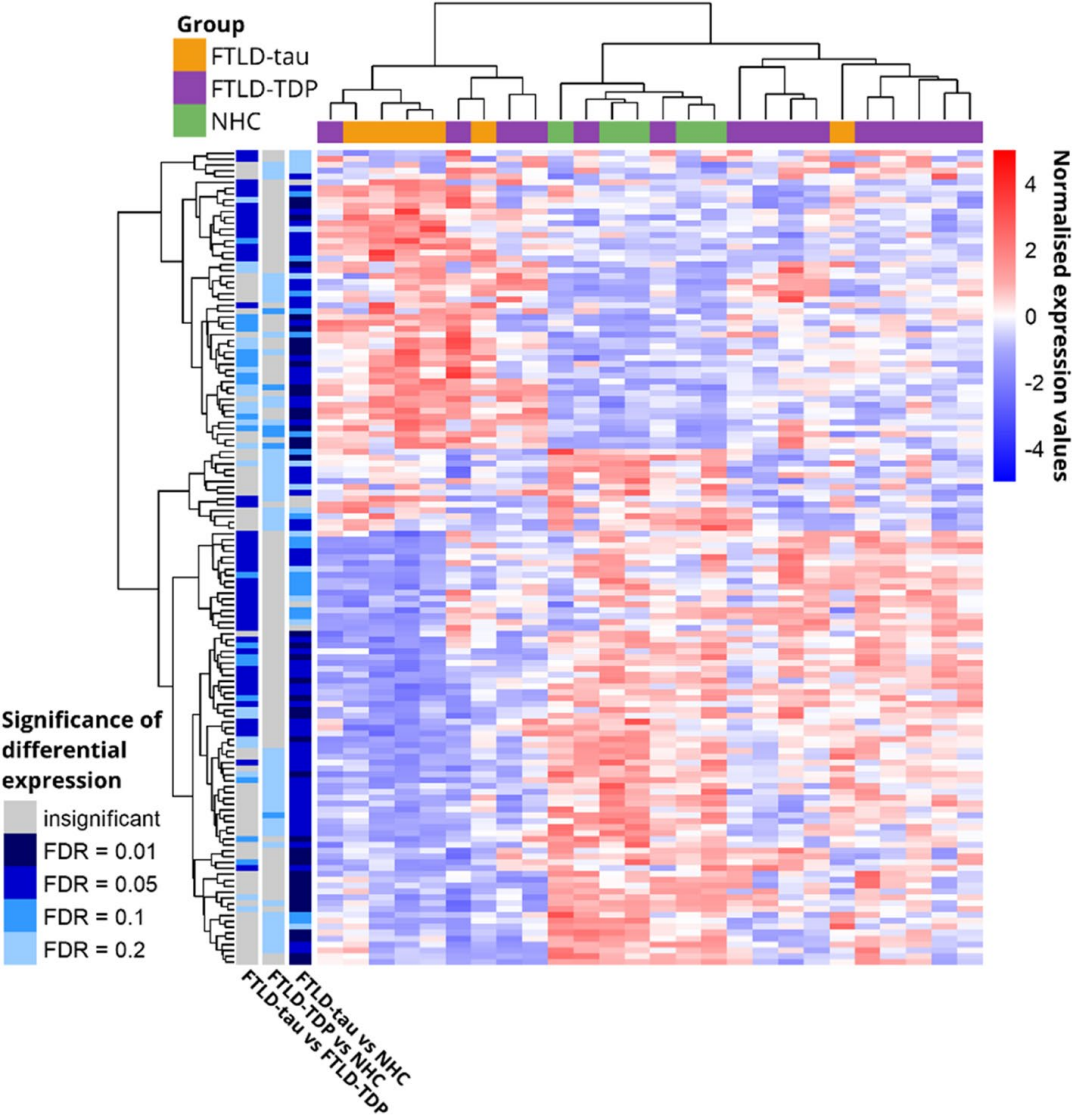
Hierarchical clustering of the samples of the discovery cohort based on the abundance levels of the 50 most significantly differentially expressed proteins resulting from the 3 pairwise comparisons. The samples are represented on the *x*-axis, FTLD-tau samples in yellow, FTLD-TDP in purple, and NHC in green. Normalized protein abundance levels are represented on the *y*-axis. Shades of red represent the upregulated proteins, and shades of blue represent the downregulated proteins. *p*-values for every 3 pairwise comparisons are indicated to the left of the figure. Shades of blue indicate statistically significant values: the darker, the more significant, and grey indicates non-significant *p*-values. To check the robustness in a data set with a limited sample size, we have repeated the clustering using Spearman correlation (Fig. S18). No significant differences could be observed

The goal of this work was to identify the dysregulated biological pathways associated with FTLD-tau and FTLD-TDP. From a total of 26 samples, LC-MS/MS identified 1801 unique proteins without any missing values across all samples. We compared protein abundance levels across the diagnostic groups and found that the largest and most significant contrast resulted from the FTLD-tau versus NHC comparison with 293 proteins differentially expressed at FDR = 0.05 (Fig. 2A). Sixty per cent of the proteins (*n* = 175) were downregulated in FTD-tau compared to NHC, and 35% (*n* = 83) of the 293 proteins had abundance level modifications beyond 50% of the NHC levels. Fewer proteins were differentially regulated in FTLD-TDP compared to NHC with 65 proteins significantly differentially expressed at FDR = 0.2 (Fig. 2C). Sixty-seven per cent (*n* = 44) were downregulated in FTLD-TDP compared to NHC, and 30% (*n* = 20) of the 65 proteins had abundance level modifications beyond 50% of the NHC levels. The comparison between FTLD-TDP and FTLD-tau yielded 49 differentially expressed proteins at FDR = 0.1 (Fig. 2B). Density distribution of the *p*-values for pairwise proteome comparisons are provided in Fig. S3, and the overlap in differentially abundant proteins between the three pairwise comparisons is shown in Fig. S19. Hierarchical clustering of the 50 most significantly differentially expressed proteins between each of the 3 comparisons revealed that the FTLD-tau and NHC samples formed clusters whereas the FTLD-TDP samples did not (Fig. 3). Pathologically, FTLD-TDP can be further classified into four (or five) subtypes according to the cortical layer localization of TDP43 aggregates [31, 32]. Our cohort included A (*n* = 5), B (*n* = 3), and C (*n* = 5) subtypes, the remainder of the samples (*n* = 2) being of undetermined subtype (Table S1). The cohort was not sufficiently powered to further investigate the clustering of the subtypes.

###  FTLD-tau and FTLD-TDP are enriched for proteins related to proteins associated with other neurodegenerative diseases and oxidative phosphorylation

**Fig. 4:**
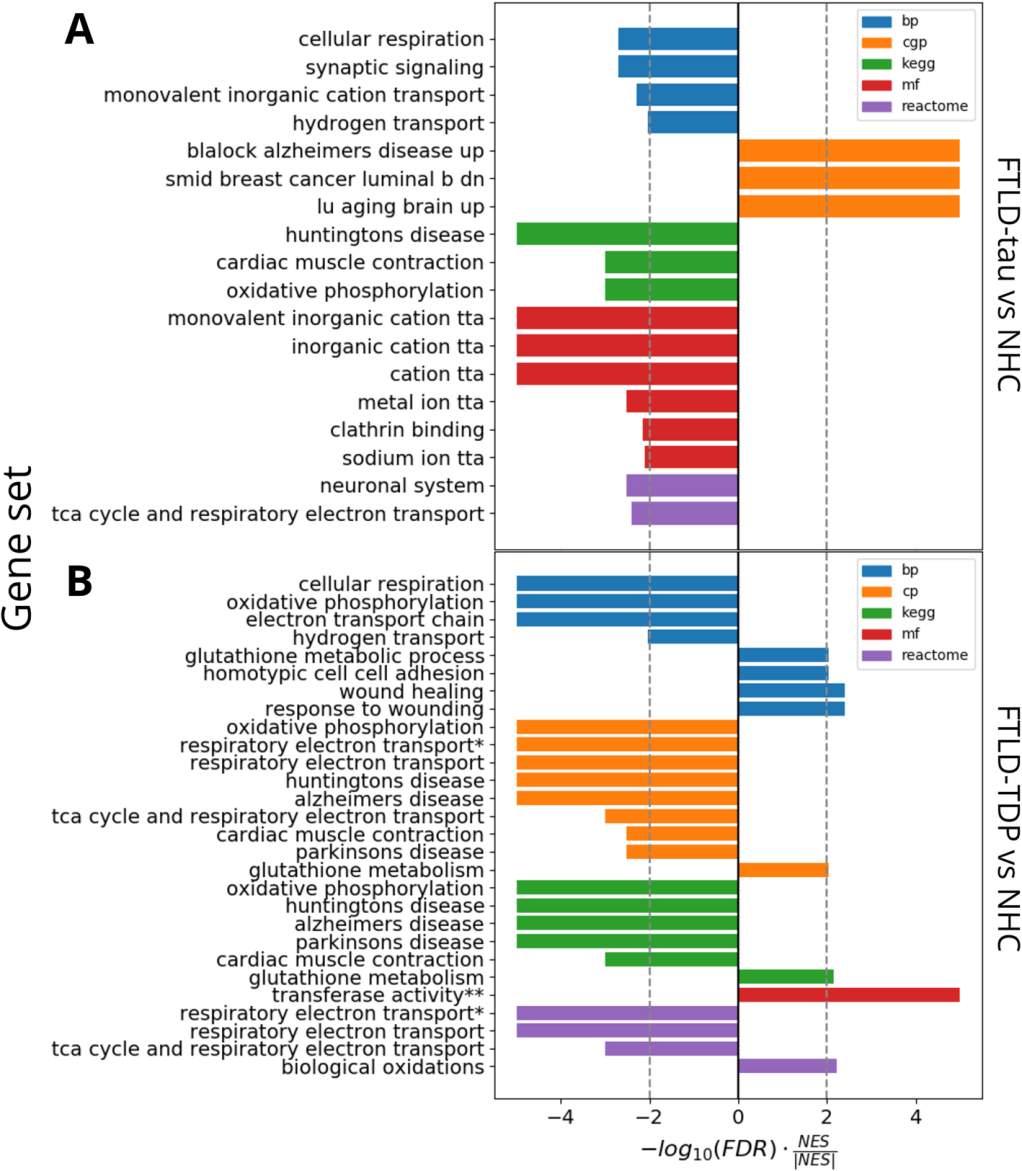
Significantly overrepresented gene sets in FTLD-tau and FTLD-TDP. Gene sets originated from 5 different databases: GO biological process database (bp, blue), chemical and genetic perturbations database (cgp, orange), Kyoto Encyclopedia of Genes and Genomes database (KEGG, green), GO molecular function database (mf, red), and REACTOME database (purple). Bars to the right indicate gene sets upregulated in FTLD-tau versus NHC (**A**) or FTLD-TDP versus NHC (**B**); bars to the left indicate gene sets downregulated in FTLD-tau versus NHC (**A**) or FTLD-TDP versus NHC (**B**). Dotted lines represent the significance threshold. The names of the gene sets are the original names as referred to in the msigdb.tta = transmembrane transporter activity. *Full name: respiratory electron transport ATP synthesis by chemiosmotic coupling and heat production by uncoupling proteins. **Full name: transferase activity transferring alkyl or aryl other than methyl groups

GSEA was performed to identify the classes of genes overrepresented in FTLD-tau and FTLD-TDP. We found that proteins upregulated in FTLD-tau were most significantly enriched for genes reported to be upregulated in Alzheimer’s disease (Fig. 4A, “blalock alzheimers disease up” gene set in the CGP database) and in the ageing frontal cortex (Fig. 4A, “lu ageing brain up” gene set in the CPG database). Proteins downregulated in FTLD-tau were most significantly enriched for genes reported to be dysregulated in Huntington’s disease (Fig. 4A, “huntingtons disease” gene set in the KEGG database), genes involved in monovalent inorganic cation transmembrane transport (Fig. 4A “monovalent inorganic cation tta” gene set in the MF database and “monovalent inorganic cation transport” gene set in the BP database), genes involved in synaptic signalling (Fig. 4A “synaptic signalling” gene set in the BP database), and genes involved in oxidative phosphorylation (Fig. 4A “cellular respiration” gene set in the BP database; “oxidative phosphorylation” gene set in the KEGG database; “tca cycle and respiratory electron transport” gene set in the REACTOME database). Proteins downregulated in FTLD-TDP were most significantly enriched for genes reported to be involved in Alzheimer’s disease (Fig. 4B “alzheimers disease” gene sets in the CPG and KEGG databases), Parkinson’s disease (Fig. 4B “parkinsons disease” gene sets in the CGP and KEGG databases), Huntington’s disease (Fig. 4B “huntingtons disease” gene sets in the CPG and KEGG databases), and genes involved in oxidative phosphorylation (Fig. 4B “oxidative phosphorylation”, “electron transport chain”, and “cellular respiration” gene sets in the BP database; “oxidative phosphorylation” and “respiratory electron transport” gene sets in the CPG database; “oxidative phosphorylation” gene set in the KEGG database; “respiratory electron transport” and “tca cycle and respiratory electron transport” gene sets in the REACTOME database).

### Network co-regulation analysis identifies 17 modules of highly co-regulated proteins

**Fig. 5:**
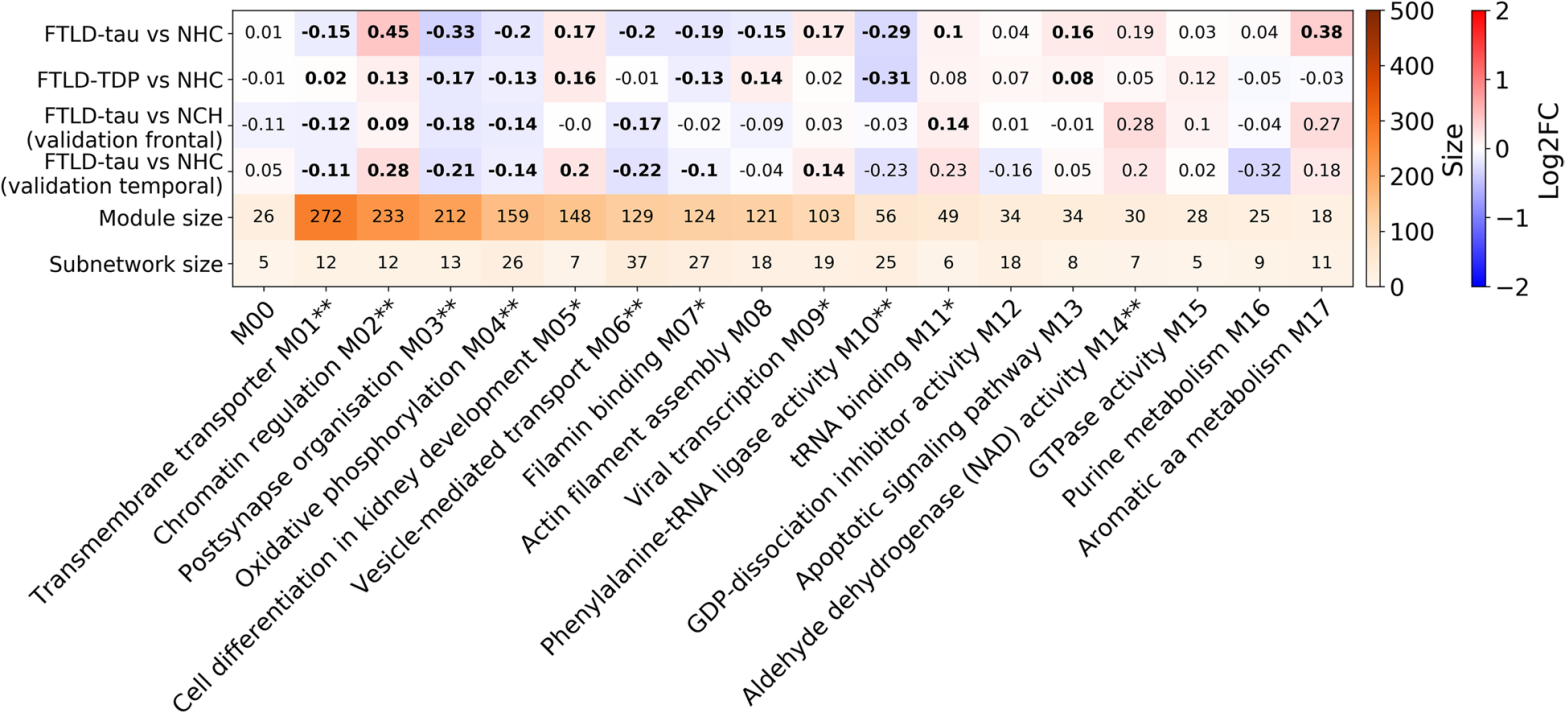
The mean differential protein abundance levels of the 17 co-regulated modules. The mean log2 fold change (log2FC) protein abundance levels for each module are provided in the table. Red indicates an upregulated mean log2FC protein abundance level within the module compared to NHC, and blue indicates a downregulated mean log2FC protein abundance level within the module compared to NHC. A bold font indicates that the mean log2FC was significantly different from zero (corrected *p* < 0.05). Additionally, the sizes of the modules and most significant subnetworks are provided. M0 is not a module but consists of the leftover proteins that do not fit in any of the 17 modules (M1 to M17). One asterisk next to a module name indicates that protein abundance patterns with the module were validated in one of the validation data sets, and two asterisks indicate that module protein abundance patterns were validated in both validation data sets. More detail about the module validation can be found in Table S2

To identify functional groups of proteins that are similarly affected in the disease, the 1801 unique proteins identified through LC-MS/MS were used to generate a protein co-expression network using the WGCNA algorithm across the total cohort [16]. The resulting network consisted of 17 modules of proteins with similar abundance patterns. The modules ranged in size from 272 (Fig. 5, module M01) to 18 proteins (Fig. 5, module M17)*.* GO analysis of the protein module members revealed the biological functions most significantly associated with each module (Table S2). Thirteen out of the 17 modules of co-expressed proteins were significantly dysregulated in FTLD-tau compared to NHC and 9 out of 17 in FTLD-TDP compared to NHC (Fig. 5). The mean log2 fold change of modules of co-expressed proteins associated with transmembrane transporter activity (module 01), post-synapse organization (module 03), oxidative phosphorylation (modules 04), filamin binding (modules 07), and phenylalanine t-RNA ligase activity (module 10) were significantly downregulated in both FTLD-tau and FTLD-TDP compared to NHC (Fig. 5). The mean log2 fold change of modules of co-expressed proteins associated with chromatin regulation (module 02), protein-containing complex binding (module 05) and apoptotic signalling (module 13) were significantly upregulated in both FTLD-tau and FTLD-TDP compared to NHC (Fig. 5). The mean log2 fold change of modules of co-expressed proteins associated with vesicle-mediated transport (M06), co-translational protein targeting to the membrane (M09), tRNA binding (M11), and oxidoreductase activity (M17) were dysregulated in FTD-tau compared to NHC (Fig. 5).

**Fig. 6:**
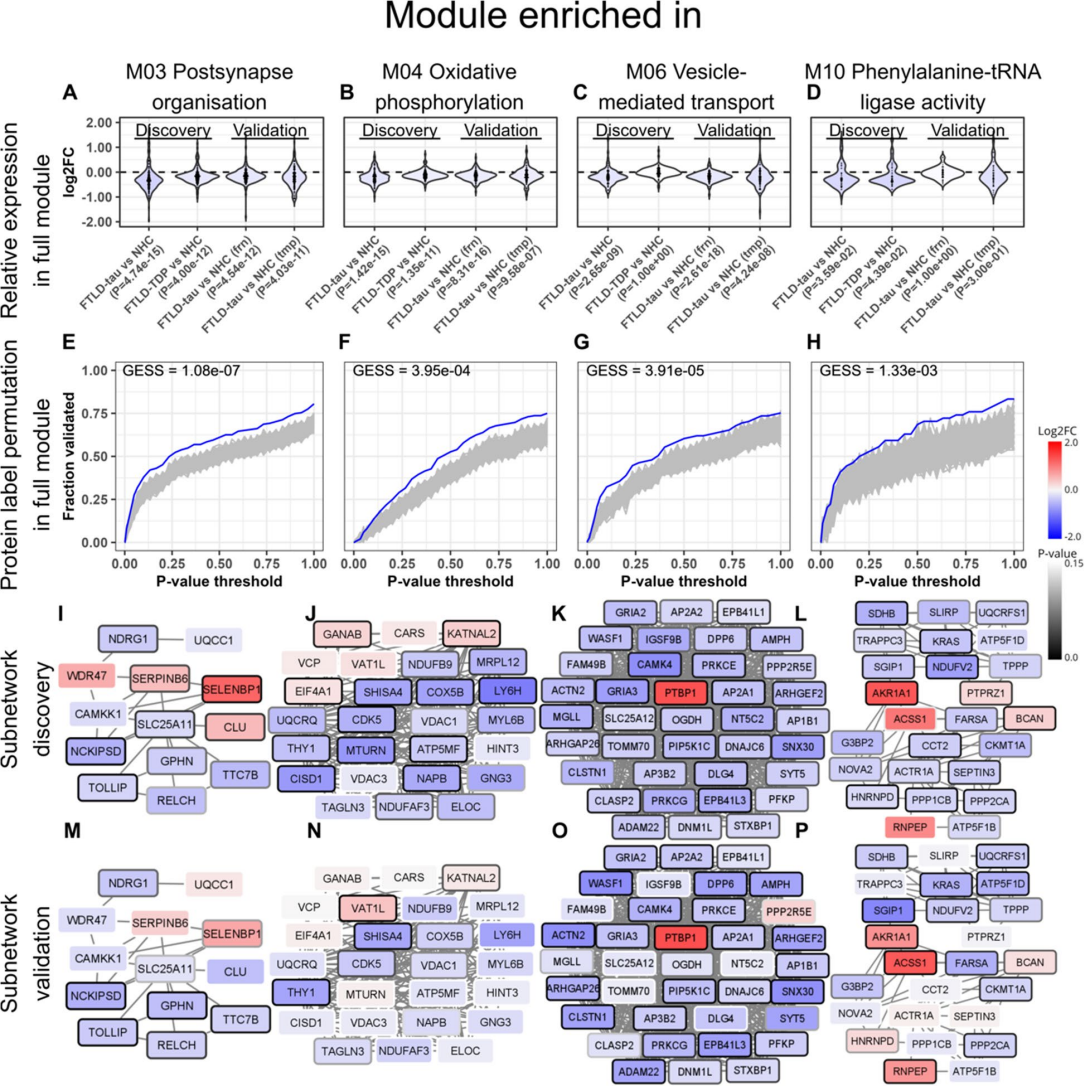
Modules and subnetworks of co-regulated proteins that are dysregulated in FTLD-tau and FTLD-TDP. **A**–**D** Density curves of the log2 fold change (log2FC) values of all the proteins belonging to a module in the discovery data set and the validation data set. The *p*-value indicates the significance of the median log2FC difference compared to NHC. **E**–**H** Permutation test to validate the differential abundance of modules identified in the discovery data set. The *y*-axis shows the fraction of proteins where the log2FC measured in the validation data set was in the same direction as in the discovery data set, and the *p*-value was below the threshold (*x*-axis) in both the discovery and validation data set (blue line). We used a permutation test, where we repeated this procedure with the protein labels in the validation data set randomly reassigned 1000× (grey lines). The module was considered validated if the average *p*-value at thresholds 0.1, 0.5, 0.5, and 0.8 was lower than 0.05 (GESS score). Fraction validated: fraction of the proteins in the module that have a log2FC in the same direction as in the discovery data set. **I**–**L** Subnetworks of most highly and significantly dysregulated proteins within the corresponding module of co-regulated proteins in the discovery data set. Red nodes indicate a positive log2FC, and blue nodes indicate a negative log2FC. The darkness of the borders reflects the significance. **M**–**P** Subnetworks of most highly and significantly dysregulated proteins in the validation data set. Red nodes indicate a positive log2FC, and blue nodes indicate a negative log2FC. The darkness of the borders reflects the significance

We next identified the subnetworks of proteins that were most highly and significantly dysregulated within each module (Figs. 5 and 6I–L). Note that the subnetworks were created based on protein abundance level correlations and log2FC values, and not on functional annotations (Fig. S1). Hence, the proteins of the subnetwork do not necessarily reflect the overall GO term(s) associated with the module to which they belong.

### Dysregulation of modules of co-regulated proteins are validated in two data sets from independent FTLD-tau tissue cohort

We next sought to validate the protein abundance patterns within modules and subnetworks of most highly and significantly dysregulated proteins within each module in two data sets of two different brain areas originating from an independent cohort (Table S1, validation cohort), focussing on FTLD-tau which showed the strongest protein abundance contrast compared to NHC. Of the 1801 proteins of the discovery data set, 1738 were also present in the frontal cortex validation data set and 1671 in the temporal cortex validation data set. To quantify the overlap in protein abundance dysregulation within each module between the discovery and validation (frontal or temporal) data sets, we devised a GESS score (see the “Materials and methods” section and Fig. 6E–H). Of the 13 modules of co-expressed proteins dysregulated in FTLD-tau, the protein abundance patterns of 9 modules were validated in one or both validation data sets (Table S2, Fig. 5), including modules 03, 04, 06, and 10 (Fig. 6).

To investigate the role of the modules in disease, we consider a module dysregulated if the distribution of log2 fold change values within that module is significantly different from zero (see the “Materials and methods” section). The mean log2 fold change protein abundance level of module 03 was downregulated in FTLD-tau compared to NHC (Fig. 6A) and consisted of 212 co-regulated proteins most significantly enriched in proteins involved in the post-synapse organisation (Fig. 5). Subnetwork analysis revealed that the most highly and significantly differentially abundant proteins associated with FTLD-tau consisted of 13 proteins among which was selenium-binding protein 1 (SELENBP1), a protein involved in ubiquitination/deubiquitination-mediated protein degradation [33], which was highly upregulated in FTLD-tau (Fig. 6I, M).

The mean log2 fold change protein abundance level of module 04 was also downregulated in FTLD-tau (Fig. 6B) and consisted of 159 proteins most significantly enriched in proteins involved in oxidative phosphorylation (Fig. 5). Subnetwork analysis revealed that the most highly and significantly dysregulated proteins consisted of 26 proteins, among which cyclin-dependent kinase 5 (CDK5), a protein involved in the phosphorylation of tau (Fig. 6J, N) [34].

The mean log2 fold change protein abundance level of module 06 was also downregulated in FTLD-tau (Fig. 6C) and consisted of 129 proteins most significantly enriched in proteins involved in vesicle-mediated transport (Fig. 5). Subnetwork analysis revealed that the most highly and significantly dysregulated proteins consisted of 37 proteins among which polypyrimidine tract-binding protein 1 (PTBP1), a splicing regulator which represses the splicing of *MAPT* exon 10, which was the only upregulated protein of the subnetwork (Fig. 6K, O).

The mean log2 fold change protein abundance level of module 10 was likewise downregulated in FTLD-tau (Fig. 6D) and consisted of 56 proteins most significantly enriched in proteins involved in phenylalanine-tRNA ligase activity (Fig. 5). Subnetwork analysis revealed that the most highly and significantly associated proteins consisted of 37 proteins among which was aldo-keto reductase family 1 member A1 (AKRA1A), a detoxifying enzyme involved in the reduction of a range of toxic aldehydes which was highly upregulated (Fig. 6L, P). The protein abundance patterns in the 4 modules and subnetworks discussed in Fig. 6 were validated in both the frontal and temporal cortex data sets. The other (validated and non-validated) modules of co-regulated proteins and subnetworks are detailed in Figs. S4-S16.

### Expression-weighted cell type enrichment analysis

**Fig. 7:**
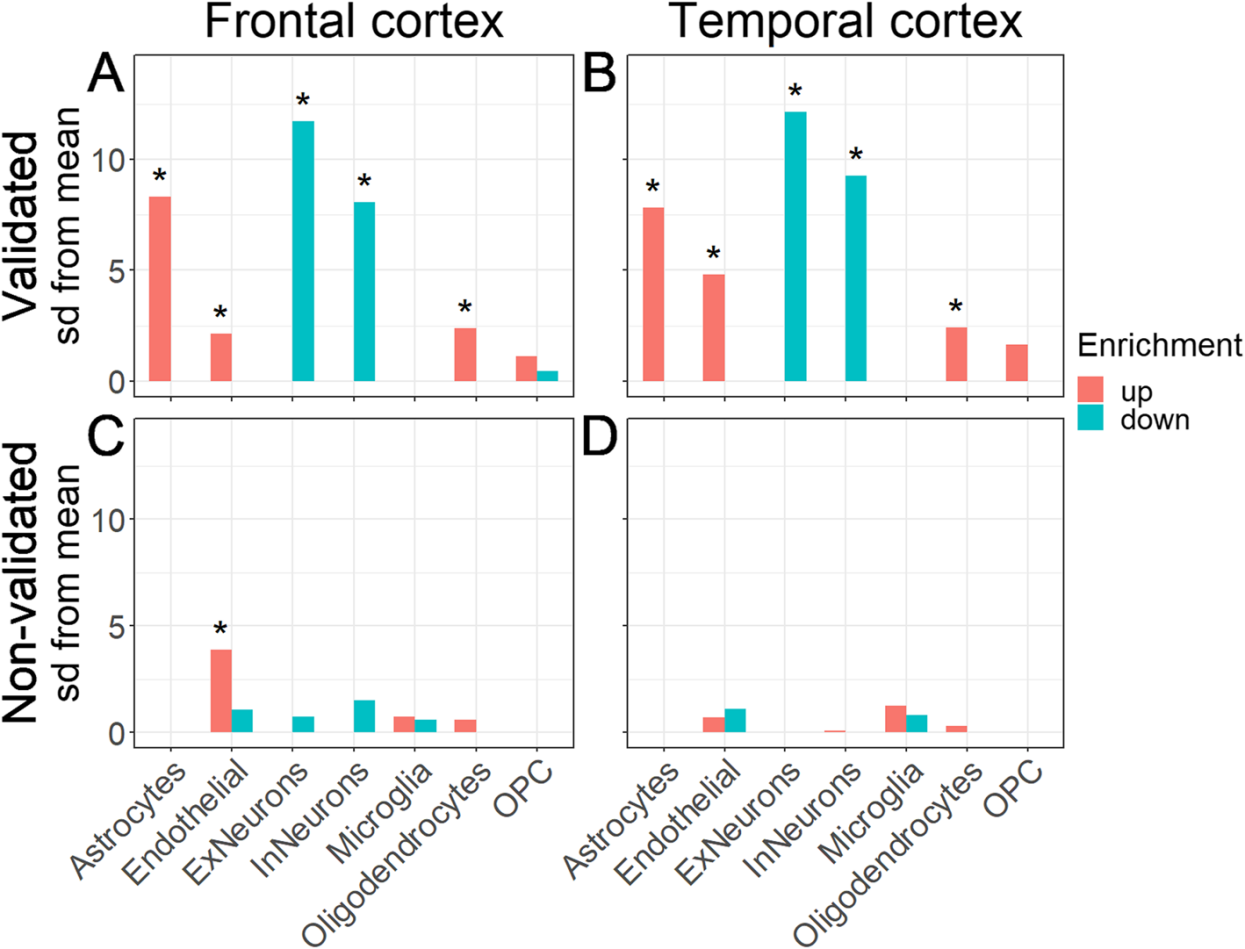
Cell type enrichment of the modules with (non-)validated protein abundance patterns. **A**, **B** The proteins in the modules that were validated were analysed for enrichment of known cell type markers (see the “Materials and methods” section). “Up” (red) indicates that the markers for that specific cell type had high log2FC values in the tau cohort compared to NHC, suggesting enrichment of these cell types in the tau subjects. “Down” (blue) indicates that the markers for that specific cell type had low log2FC values in the tau cohort compared to NHC, suggesting depletion of these cell types in the tau subjects. This analysis was performed both on the modules validated in the frontal samples (**A**) and on the modules validated in the temporal samples (**B**) of the validation set. **C**, **D** The same procedure was performed on the modules where we were not able to validate the protein abundance patterns

**Fig. 8:**
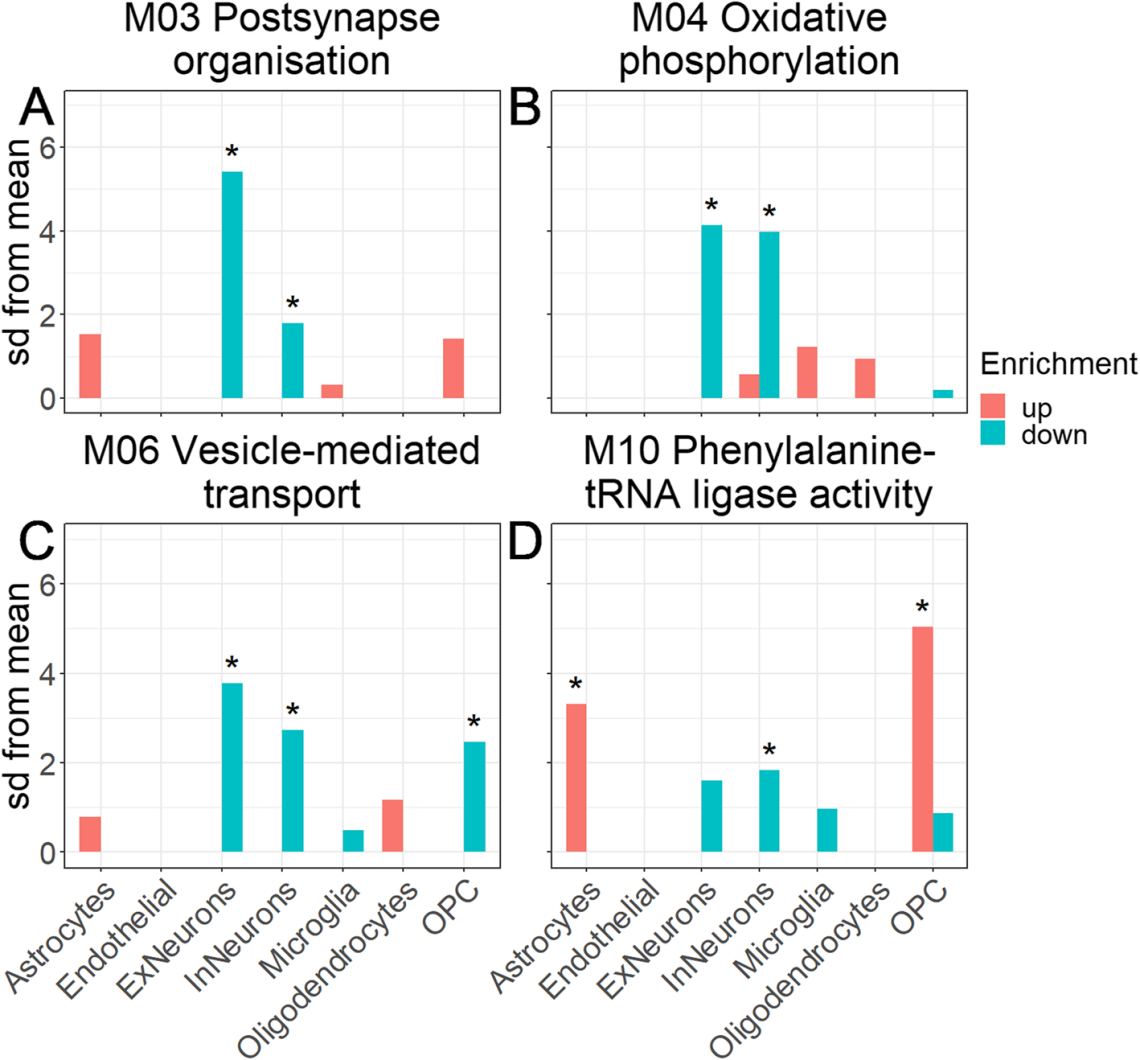
Cell type enrichment in modules **A** M03 (postsynapse organisation), **B** M04 (oxidative phosphorylation), **C** M06 (vesicle-mediated transport), and **D** M10 (phenylalanine-tRNA ligase activity). Cell type enrichment in each of the modules was determined based on the log2FC values of specific CNS cell-type protein markers (shown here for the temporal validation set). Included cell types are astrocytes, endothelial cells, excitatory neurons, inhibitory neurons, microglia, oligodendrocytes, and oligodendrocyte precursor cells. Enrichment up (red bar) means that the markers for that cell type had high log2FC values in the FTLD-tau samples compared to the control group, and enrichment down (blue bar) means that the markers for that cell type had low log2FC values in the FTLD-tau samples compared to the control group

To determine which cell types are driving the abundance changes in each co-regulated module of proteins, we evaluated the enrichment of specific cell type marker proteins in each module (see the “Materials and methods” section). We found that in FTLD-tau, the modules with validated differential abundance were associated with higher abundance levels of astrocyte, endothelial cell, and (only in the temporal cortex) OPC marker proteins and lower abundance levels of excitatory and inhibitory neuron marker proteins (Fig. 7A, B). In contrast, the non-validated modules were not associated with any cell-type marker proteins (Fig. 7C, D). When considering the changes per module (Fig. 8), proteins associated with astrocytes and OPCs were upregulated in FTLD-tau compared to NHC in the module associated with phenylalanine-tRNA ligase activity (M10), indicating that this mechanism is specifically affected in OPCs and not the other cell types (Fig. 8). In contrast, proteins associated with excitatory and inhibitory neurons were downregulated in FTLD-tau compared to NHC in modules associated with post-synapse organisation (M03), oxidative phosphorylation (M04), and vesicle-mediated transport (M06), indicating protein abundance dysregulation in these processes are mostly affected in neurons (Fig. 8). An overview of cell-type enrichment of all modules is given in Fig. S17.

## Discussion

### FTLD-tau has a clearer pathological signature than FTLD-TDP

This study aimed to identify and validate dysregulated proteins and biological pathways and the cell types driving these changes associated with FTLD-tau or FTLD-TDP, using a systems-level proteomics approach applied on brain cortex tissue. We found that the middle frontal gyrus cortical proteome was most significantly altered in FTLD-tau compared to age-matched NHC. The proteomic changes in FTLD-TDP were more heterogeneous, resulting in a weaker contrast with NHC. Weighted co-expression network analysis revealed that thirteen modules of co-regulated proteins were dysregulated in FTLD-tau and/or FTLD-TDP. Dysregulation of 9 of these modules was confirmed in 2 independent validation data sets of FLTD-tau and control temporal and frontal cortex tissue (total *n* = 24). We validated oxidative phosphorylation, scavenger mechanisms, chromatin dysregulation, and clathrin-mediated transport as key dysregulated pathways in both the frontal and temporal cortices of FTLD-tau. Dysregulated modules were associated with changes in astrocyte and endothelial cell protein abundance levels, indicating pathological changes in FTD are not exclusively driven by neurons.

We hypothesized the relatively fewer changes in FTLD-TDP result from the higher pathological heterogeneity of FTLD-TDP, which can be further classified into four (or five) subtypes according to the cortical layer localization of TDP43 aggregates [31, 32]. Conceptually similar findings were reported by our group when comparing the ante mortem cerebrospinal fluid (CSF) proteome of FTLD-TDP and FTLD-tau individuals with that of NHC, the latter comparison yielding many more differentially regulated proteins than the former [35]. To date, all CNS tissue proteomic studies investigated FTLD-TDP cases as one group, so that our findings cannot be corroborated yet [36–39].

This study was performed on a relatively small number of subjects (*n* = 26 for the discovery cohort and *n* = 24 for the validation cohort). Hence, this study has too few samples to further stratify the FTLD-TDP samples into these subgroups with sufficient statistical power. Human brain tissue is difficult to obtain, and because FTD is a rare disease, cohorts tend to be rather small. With a larger sample size, more complex co-regulation patterns linked to FTLD-TDP subtypes might also be unravelled. The strength of our results lies in that they were validated in an independent cohort.

### Pathways related to tau phosphorylation and alternative splicing affecting tau aggregation propensity affected in FTLD-tau

Several tau-related proteins were dysregulated in the FTLD-tau data set compared to NHC, including PTBP1, a splicing regulator which represses the splicing of *MAPT* exon 10 [40]. This is relevant to FTLD-tau pathology as alternative splicing of exon 10 results in tau isoforms containing either three or four microtubule-binding repeats (3R-tau and 4R-tau, respectively). The difference in the number of repeats determines the strength of the binding of tau to microtubules, thus modulating the stability of tau and its propensity to aggregation [41, 42]. Increased levels of PTBP1 induce splicing of exon 10, which favours the less aggregation-prone 3R-tau isoform. CDK5 was among the most significantly downregulated proteins of the M04 subnetwork (Fig. 6). CDK5 is a proline-directed serine/threonine kinase involved in tau phosphorylation via the regulation of microtubule affinity-regulating kinase 4 (MARK4) activity [43], and its aberrant activation leads to tau aggregation and neurodegeneration [44]. We hypothesize that increased PTBP1 and decreased CDK5 abundance represent compensatory mechanisms in the face of tau aggregation and could thus be interesting therapeutic targets. Other tau-related proteins upregulated in FTLD-tau included protein S100 A1, involved in the regulation of tau phosphorylation [45], plasma membrane-binding protein ANXA2, a tau-binding protein that contributes to the enrichment of tau in the axon [46, 47], microtubule-associated protein (MAPRE1), and tubulin polymerization-promoting protein family member 3 (TPPP3).

### Overlap between proteins most significantly deregulated in FTLD-TDP in our data set and other studies suggest possible FTLD-TDP biomarker candidates

A smaller number of differentially regulated proteins was identified in the FTLD-TDP frontal cortex proteome compared to that of NHC. However, four proteins among the most highly upregulated proteins in FTLD-TDP and five proteins among the most downregulated proteins in FTLD-TDP have been reported to be dysregulated in the FTLD-TDP frontal cortex in two separate independent proteomic studies, supporting our findings (confirmed upregulated proteins: biliverdin reductase B (BLVRD), chloride intracellular channel 4 (CLIC4), haemoglobin subunit delta (HBD), platelet-activating factor acetyl hydrolase 1b catalytic subunit 3 (PAFAH1B3); downregulated proteins: AP2 associated kinase 1 (AAK1), NADH ubiquinone oxidoreductase subunit A2 (NDUFA2), N-terminal EF-hand calcium-binding protein 1 (NECAB1), protein kinase C and casein kinase substrate in neurons 1 (PACSIN1), proline-rich transmembrane protein 2 (PRRT2)) [14, 36, 37].

### GSEA and co-expression analysis reveal the biological pathways affected in FTLD-tau and FTLD-TDP

Both FTLD-tau and FTLD-TDP data sets were enriched for gene sets previously associated with Alzheimer’s disease and Huntington’s disease, suggesting dysregulated pathways common to neurodegenerative conditions, next to gene sets associated with oxidative phosphorylation (Fig. 4). Impaired oxidative phosphorylation is a common feature of many neurodegenerative conditions [48]. In particular, tau aggregates have been reported to directly disrupt mitochondrial function [49].

To identify the groups of co-regulated proteins beyond the currently described pathways, we designed a novel workflow (Fig. S1), which resulted in the identification of seventeen modules of co-regulated proteins across the total cohort, 13 of which were dysregulated in FTLD-tau and/or FTLD-TDP. Dysregulation of 9 of these modules was validated in frontal and/or temporal cortex data sets derived from an independent validation cohort of FTLD-tau and NHC. A module enriched for proteins involved in oxidative phosphorylation (M04) was downregulated in FTLD-tau and FTLD-TDP compared to NHC, further underscoring the importance of impaired mitochondrial function in FTLD similar to other neurodegenerative conditions. Oxidative phosphorylation generates reactive oxygen species that can damage cells when produced in excess and that are mitigated by the expression of scavengers. We found that modules of co-regulated proteins associated with aldehyde dehydrogenase activity (M14), regulation of superoxide metabolic process (M16), and oxidoreductase activity (M17), all of which are important cellular defence mechanisms against oxidative stress, were upregulated in FTLD-tau compared to NHC. These findings indicate tentative compensatory mechanisms may persist late in the disease course and that sustaining scavenger systems is an interesting therapeutic approach. A module of co-regulated proteins enriched for proteins involved in the regulation of vesicle-mediated transport (M06) was downregulated in FTLD-tau compared to NHC. Clathrin-mediated endocytosis is a key process in vesicular trafficking that transports a wide range of cargo molecules from the cell surface to the interior, including synaptic vesicle recycling [50]. Clathrin-mediated endocytosis is also linked to the endo-lysosomal degradative pathway and proteostasis network, which consists of membranous organelles specialized in regulating both intracellular trafficking and proteostasis. Recent in vitro results revealed that similar to other toxic protein aggregates, tau aggregation inhibits the chaperone-regulated proteostasis network processes of clathrin-mediated vesicular trafficking and protein folding in the cytoplasm [51]. In addition, neural stem cells derived from patients with an N279K tau mutation displayed impaired endocytic trafficking as evidenced by the accumulation of endosomes and exosomes and a reduction of lysosomes [52, 53]. Together, these results suggest a key role of endosomal vesicle trafficking in FTLD-tau pathology. A module of co-regulated proteins associated with chromatin regulation (M02) was upregulated in FTLD-tau. In animal models of tauopathies, a study reported that tau promotes global heterochromatin relaxation leading to aberrant gene expression eventually resulting in neurodegeneration [54]. There is to our knowledge no study investigating whether dysregulation of heterochromatin relaxation state is at play in humans, warranting further research in this direction. Downregulation of M06, upregulation of M02, and the subnetworks of most dysregulated proteins in these modules were validated in both the FTLD-tau frontal and temporal cortex independent data sets, indicating dysregulation of these modules is not anatomically specific, but rather associated with FTLD-tau. Modules of co-regulated proteins associated with post-synapse organisation (M03), filamin binding (M07), protein translation (M09, M10, and M11), and transmembrane transport (M01) were downregulated in FTLD-tau compared to NHC, which together may reflect cellular dysfunction.

### Astrocytes and OPCs are involved in FTLD-tau

We found that in addition to neurons, cellular dysfunction is also present in other cell types such as astrocyte and OPC proteins for example M10 (Fig. 8), indicating specific roles for these cell types in driving FTD pathology. Interestingly, glial fibrillary acidic protein (GFAP), fatty acid-binding protein 7 (FABP7), hepatic and glial cell adhesion molecule (HEPACAM), and protein S100B, all four of which are overexpressed by reactive astrocytes, were among the most highly upregulated proteins in the FTLD-tau data set. This upregulation may reflect unspecific astrogliosis. However, in light of the enrichment for astrocyte proteins identified in some dysregulated modules and their essential role in the viability of neurons, this upregulation may reflect the loss of normal astrocyte function specifically contributing to FTLD-tau pathophysiology, as it has been suggested recently for Alzheimer’s disease and other neurodegenerative conditions [33, 55–57]. Most cell type-specific proteins are found in multiple modules, rather than one module specifically. This is not surprising, considering that the modules were not created based on these cell-type annotations. These findings indicate that cell types might be affected in multiple ways, also indicated by the different GO terms associated with each module that these proteins are found in. However, neurons specifically have a consistently lower abundance in FTLD-tau compared to the controls in most modules, suggesting that this is a signature of neuronal degradation typical of brain disease.

## Conclusions

This study confirms the importance of oxidative phosphorylation and scavenger mechanisms dysfunction in FTLD-tau and identifies chromatin dysregulation and clathrin-mediated transport as key dysregulated pathways in FTLD-tau, both in the frontal and temporal cortices. Tau-related proteins were identified as highly and significantly dysregulated proteins within module subnetworks and may thus be considered as therapeutic targets. The dysregulated modules of co-expressed proteins were explicitly associated with changes in neuronal, astrocyte, and OPC protein abundance levels, indicating pathological changes are not exclusively driven by neurons. Future research should study these identified proteins and their role in FTLD-tau in more detail.

## Supplementary Information

**Additional file 1: Fig. S1.** Co-expression analysis and submodule identification workflow. Proteins were clustered into modules based on expression level correlations. GO term enrichment analysis was used to identify the molecular function most significantly enriched within each module of co-regulated proteins. Within each module, a submodule of most highly and significantly differentially regulated proteins was identified. **Fig. S2.** Workflow for module validation. For each module, the log2FC expression values of FTLD-tau versus NHC calculated in the discovery dataset was evaluated in the validation data set. The fraction of proteins that was dysregulated in the same direction in both datasets and that had a p-value lower than a certain threshold in both datasets was calculated. Subsequently, the protein labels in the validation data set were shuffled and the same fraction was calculated. This permutation was repeated 1,000 times. If the fraction of proteins was significantly higher in the comparison with the correct labels than in the comparison with the permutated labels (GESS score (see Methods) taken as the average of the P-values at thresholds 0.1, 0.2, 0.5, 0.8), the module was considered validated. **Fig. S3.** Density distribution of the P-values (x-axis) for pairwise proteome comparisons. (**A**) FTLD-tau versus NHC; (**B**) FTLD-tau versus FTLD-TDP; (**C**) FTLD-TDP versus NHC. A beta-uniform mixture (BUM) model was fitted (red and curved blue lines) to the distributions to determine the P-value thresholds required for different FDR values (vertical black lines are used to indicate the thresholds at FDR=0.1). **Figs. S4-S16.** Modules and subnetworks of co-regulated proteins that are dysregulated in FTLD-tau and FTLD-TDP for each of the modules not discussed in the main manuscript. **A** Density curves of the log2 fold-change (log2FC) values of all the proteins belonging to a module in the discovery data set and the validation data set. The p-value indicates the significance of the median log2FC difference compared to NHC. **B** Permutation test to validate the modules identified in the discovery data set. A module was validated if the log2FC measured in the validation data set was in the same direction as in the one measured in the discovery data set, and if the p-value was below the threshold (blue line) in both the discovery and validation data sets. We used a permutation test, where we repeated this procedure with the protein labels in the validation data set randomly reassigned 1,000x (gray lines). The module was considered validated if the average p-value at thresholds 0.1, 0.5, 0.5, and 0.8 was lower than 0.05 (GESS score). Fraction validation: fraction of the proteins in the module that have a log2FC in the same direction as in the discover data set. **C** Subnetworks of most highly and significantly dysregulated proteins within the corresponding module of co-regulated proteins in the discovery data set. Red nodes indicate a positive log2FC and blue nodes a negative log2FC. The darkness of the borders reflects the significance. **D** Subnetworks of most highly and significantly dysregulated proteins in the validation data set. Red nodes indicate a positive log2FC and blue nodes a negative log2FC. The darkness of the borders reflects the significance. **Fig. S17.** Cell type enrichment in all modules. Cell type enrichment in each of the modules was determined based on the log2FC values of specific CNS cell type protein markers. Included cell types are astrocytes, endothelial cells, excitatory neurons, inhibitory neurons, microglia, oligodendrocytes and oligodendrocyte precursor cells. Enrichment up (red bar) means that the markers for that cell type had high log2FC values in the FTLD-tau samples compared to the control group, enrichment down (blue bar) means that the markers for that cell type had low log2FC values in the FTLD-tau samples compared to the control group. **Fig. S18.** Hierarchical clustering of the samples of the discovery cohort based on the abundance levels of the 50 most significantly differentially expressed proteins resulting from the 3 pairwise comparisons using Spearman correlation. The samples are represented on the x-axis, FTLD-tau samples in yellow, FTLD-TDP in purple, and NHC in green. Normalised protein abundance levels are represented on the y-axis. Shades of red represent upregulated proteins, and shades of blue downregulated proteins. P-values for each 3 pairwise comparisons are indicated to the left of the figure. Shades of blue indicate statistically significant values; the darker the more significant, and gray indicates non-significant p-values. **Fig. S19.** Overlap in significant proteins between the different pairwise comparisons. A significance cutoff at FDR=0.1 was used here. The figure shows that most proteins significantly different in FTLD-TDP vs NHC and FTLD-tau vs FTLD-TDP are also significant in FTLD-tau vs NHC. There are 12 proteins significantly different between FTLD-tau and FTLD-TDP that are not captured in FTLD- tau vs NHC. **Table S1.** Discovery and validation cohorts. mfg, medial frontal gyrus; temp, temporal lobe; NHC, neurologically healthy control; AD, autosomal dominant; S, sporadic; Fa, familial; NA, not applicable; f, female; m, male; PMI, postmortem interval in hours and minutes. Age is in years. **Table S2.** Module summary. Module identity (number), module size and largest submodule size (number of proteins in the module/submodule), and validation in the FTLD-tau medial frontal gyrus cortex or temporal cortex data set of the validation cohort. Module function indicates the Gene ontology (GO) term enrichment analysis biological function most significantly associated with the module. The last column indicates the number of proteins in the subnetwork that are associated with the GO term listed in the column “Module function”. **Table S3.** Mean age, PMI and gender distribution in the three pathological groups. No significant differences in these variables were found between the three groups.

## Data Availability

Data and scripts are available on GitHub: https://github.com/ibivu/FTD_PRODIA. Apart from the GSEA, all analyses were performed using R version 3.6.1 and python version 2.7.15.

## Abbreviations

C90RF: Chromosome 9 open reading frame gene
CGP: Chemical and genetic perturbations
CNS: Central nervous system
EWCE: Expression-weighted cell type enrichment analysis
FDR: False discovery rate
FTLD: Fronto-temporal lobar degeneration
fFTLD: Familial FTLD
GO: Gene Ontology
GRN: Progranulin gene
GSEA: Gene set enrichment analysis
KEGG: Kyoto Encyclopedia of Genes and Genomes
LC-MS/MS: Liquid chromatography tandem mass spectrometry
MAPT: Microtubule-associated protein tau gene
NES: Normalized Enrichment Scores
NHC: Neurologically healthy controls
OPC: Oligodendrocyte progenitor cell
SWATH: Sequential window acquisition of all theoretical mass spectra
TARDB: TDP43 gene
TDP43: Transactive response DNA-binding protein 43 kDa
VCP: Valosin-containing protein
WGCNA: Weighted gene coexpression network analysis
