# Supplementary material for: Clusters of co-abundant proteins in the brain cortex associated with fronto-temporal lobar degeneration
Source: Alzheimers Res Ther. 2023 Mar 23;15:59. doi: 10.1186/s13195-023-01200-1 (PMC10035199; doi:10.1186/s13195-023-01200-1)
Supplement: Supplementary file 1 — Additional file 1: Fig. S1. Co-expression analysis and submodule identification workflow. Proteins were clustered into modules based on expression level correlations. GO term enrichment analysis was used to identify the molecular function most significantly enriched within each module of co-regulated proteins. Within each module, a submodule of most highly and significantly differentially regulated proteins was identified. Fig. S2. Workflow for module validation. For each module, the log2FC expression values of FTLD-tau versus NHC calculated in the discovery dataset was evaluated in the validation data set. The fraction of proteins that was dysregulated in the same direction in both datasets and that had a p-value lower than a certain threshold in both datasets was calculated. Subsequently, the protein labels in the validation data set were shuffled and the same fraction was calculated. This permutation was repeated 1,000 times. If the fraction of proteins was significantly higher in the comparison with the correct labels than in the comparison with the permutated labels (GESS score (see Methods) taken as the average of the P-values at thresholds 0.1, 0.2, 0.5, 0.8), the module was considered validated. Fig. S3. Density distribution of the P-values (x-axis) for pairwise proteome comparisons. (A) FTLD-tau versus NHC; (B) FTLD-tau versus FTLD-TDP; (C) FTLD-TDP versus NHC. A beta-uniform mixture (BUM) model was fitted (red and curved blue lines) to the distributions to determine the P-value thresholds required for different FDR values (vertical black lines are used to indicate the thresholds at FDR=0.1). Figs. S4-S16. Modules and subnetworks of co-regulated proteins that are dysregulated in FTLD-tau and FTLD-TDP for each of the modules not discussed in the main manuscript. A Density curves of the log2 fold-change (log2FC) values of all the proteins belonging to a module in the discovery data set and the validation data set. The p-value indicates the significance of the [file 13195_2023_1200_MOESM1_ESM.pdf]

Supplementary figures and tables

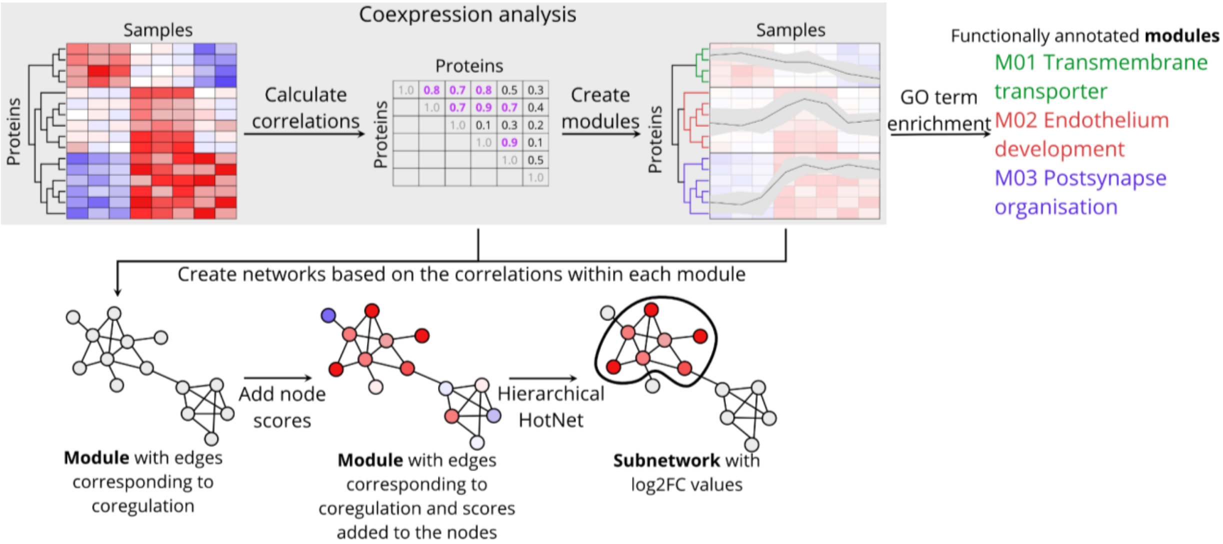

Figure S1 Co-expression analysis and submodule identification workflow.
Proteins were clustered into modules based on expression level correlations. GO term enrichment analysis was used to identify the molecular function most significantly enriched within each module of co-regulated proteins. Within each module, a submodule of most highly and significantly differentially regulated proteins was identified.

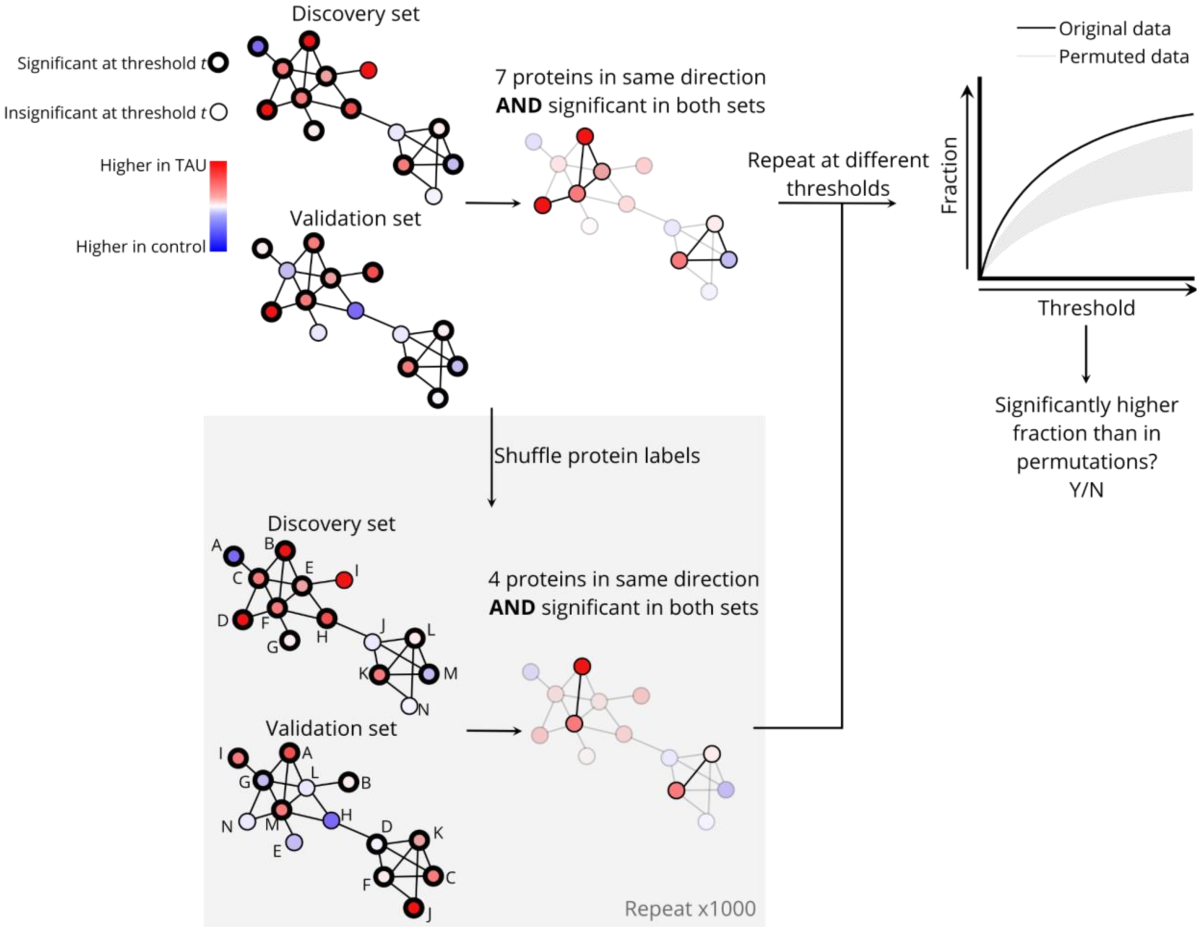

Figure S2 Workflow for module validation

For each module, the log2FC expression values of FTLD-tau versus NHC calculated in the discovery dataset was evaluated in the validation data set. The fraction of proteins that was dysregulated in the same direction in both datasets and that had a p-value lower than a certain threshold in both datasets was calculated. Subsequently, the protein labels in the validation data set were shuffled and the same fraction was calculated. This permutation was repeated 1,000 times. If the fraction of proteins was significantly higher in the comparison with the correct labels than in the comparison with the permutated labels (GESS score (see Methods) taken as the average of the P-values at thresholds 0.1, 0.2, 0.5, 0.8), the module was considered validated.

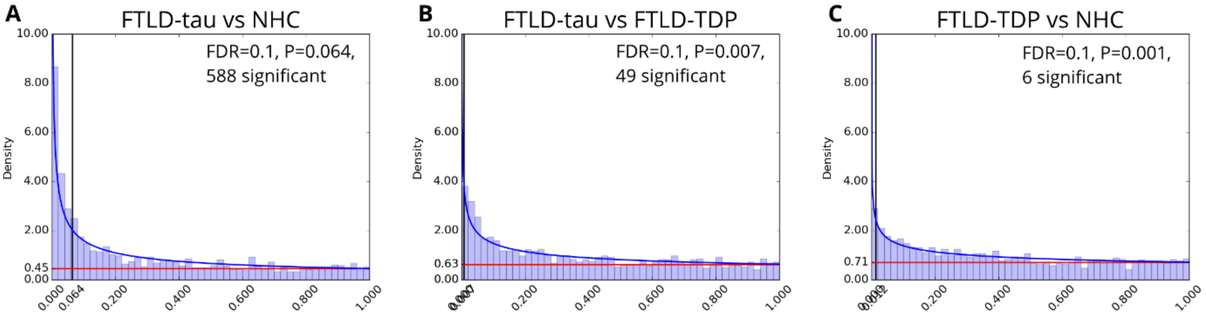

Figure S3 Density distribution of the P-values (x-axis) for pairwise proteome comparisons
(A) FTLD-tau versus NHC; (B) FTLD-tau versus FTLD-TDP; (C) FTLD-TDP versus NHC. A beta-uniform mixture (BUM) model was fitted (red and curved blue lines) to the distributions to determine the P-value thresholds required for different FDR values (vertical black lines are used to indicate the thresholds at FDR=0.1).

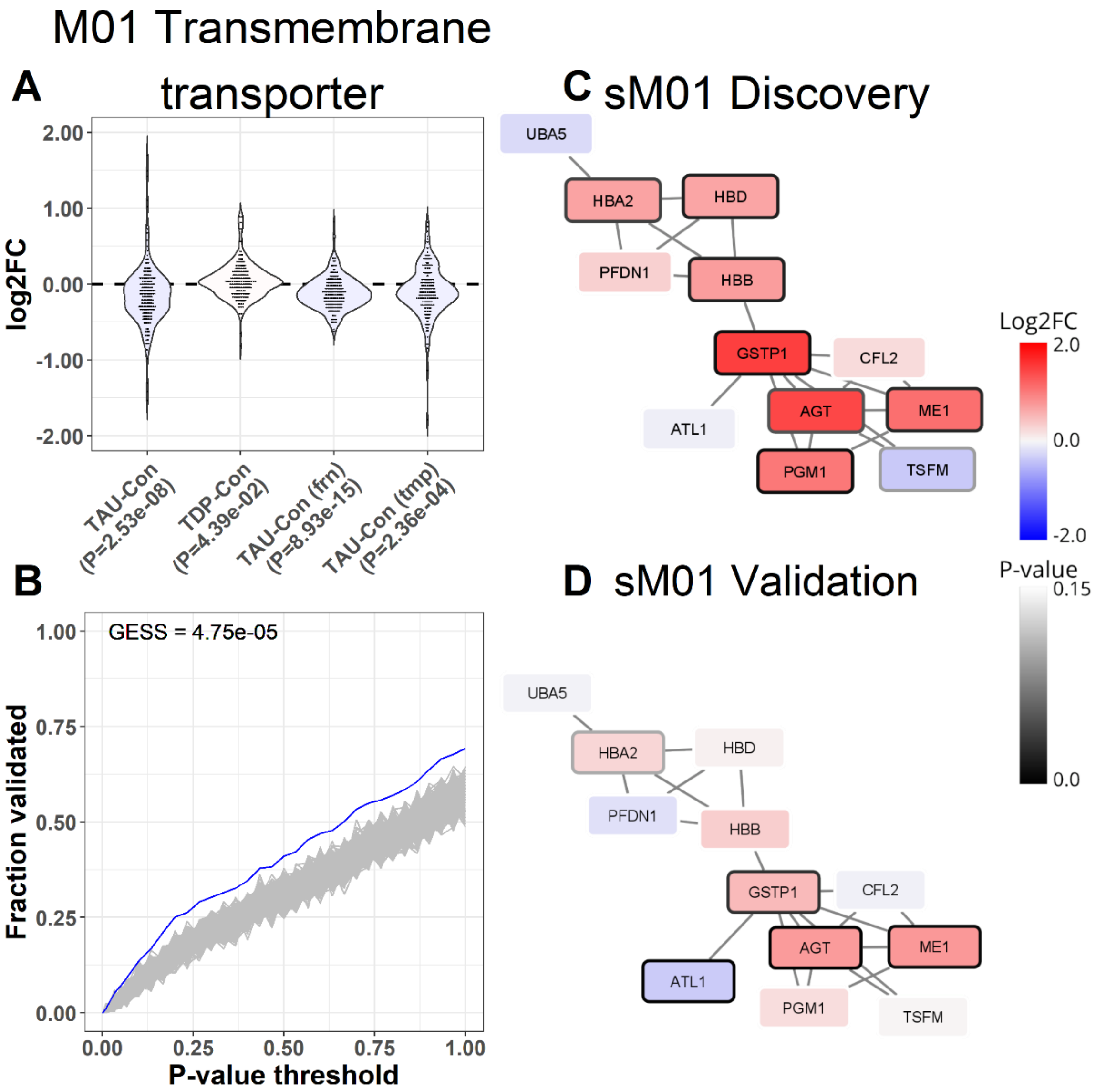

Figure S4-S16 Modules and subnetworks of coregulated proteins that are dysregulated in FTLD-tau and FTLD-TDP for each of the modules not discussed in the main manuscript. **A** Density curves of the log2 fold-change (log2FC) values of all the proteins belonging to a module in the discovery data set and the validation data set. The p-value indicates the significance of the median log2FC difference compared to NHC. **B** Permutation test to validate the modules identified in the discovery data set. A module was validated if the log2FC measured in the validation data set was in the same direction as in the one measured in the discovery data set, and if the p-value was below the threshold (blue line) in both the discovery and validation data sets. We used a permutation test, where we repeated this procedure with the protein labels in the validation data set randomly reassigned 1,000x (gray lines). The module was considered validated if the average p-value at thresholds 0.1, 0.5, 0.5, and 0.8 was lower than 0.05 (GESS score). Fraction validation: fraction of the proteins in the module that have a log2FC in the same direction as in the discover data set. **C** Subnetworks of most highly and significantly dysregulated proteins within the corresponding module of co-regulated proteins in the discovery data set. Red nodes indicate a positive log2FC and blue nodes a negative log2FC. The darkness of the borders reflects the significance. **D** Subnetworks of most highly and significantly dysregulated proteins in the validation data set. Red nodes indicate a positive log2FC and blue nodes a negative log2FC. The darkness of the borders reflects the significance.

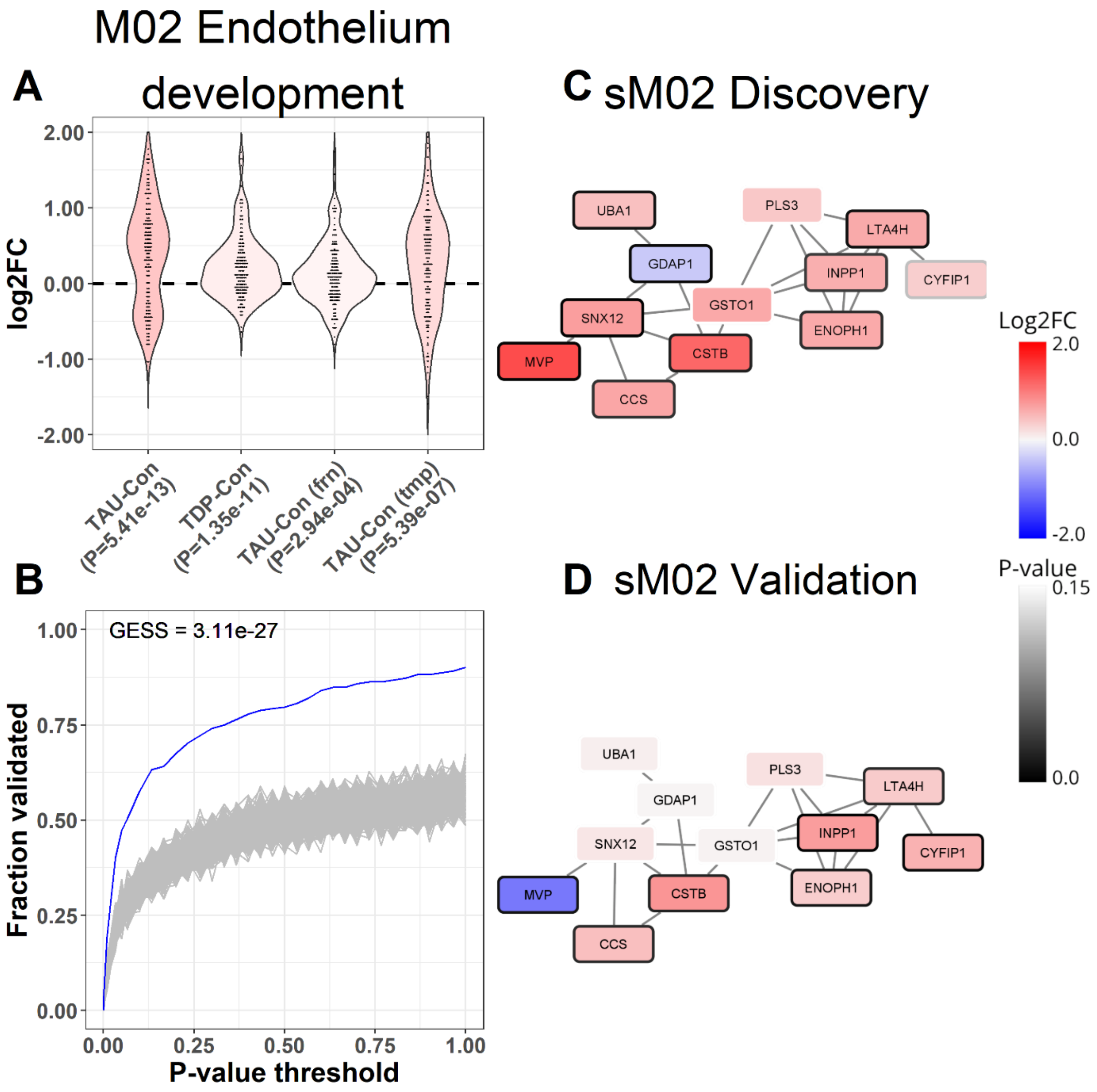

A
M02 Endothelium development
log2FC
2.00
1.00
0.00
-1.00
-2.00
TAU-Con (P=5.41e-13)
TDP-Con (P=1.35e-11)
TAU-Con (frn) (P=2.94e-04)
TAU-Con (tmp) (P=5.39e-07)
B
GESS = 3.11e-27
Fraction validated
1.00
0.75
0.50
0.25
0.00
P-value threshold
C sM02 Discovery
UBA1
PLS3
LTA4H
GDAP1
INPP1
CYFIP1
SNX12
GSTO1
ENOPH1
MVP
CSTB
CCS
Log2FC
2.0
0.0
-2.0
P-value
0.15
0.0
D sM02 Validation

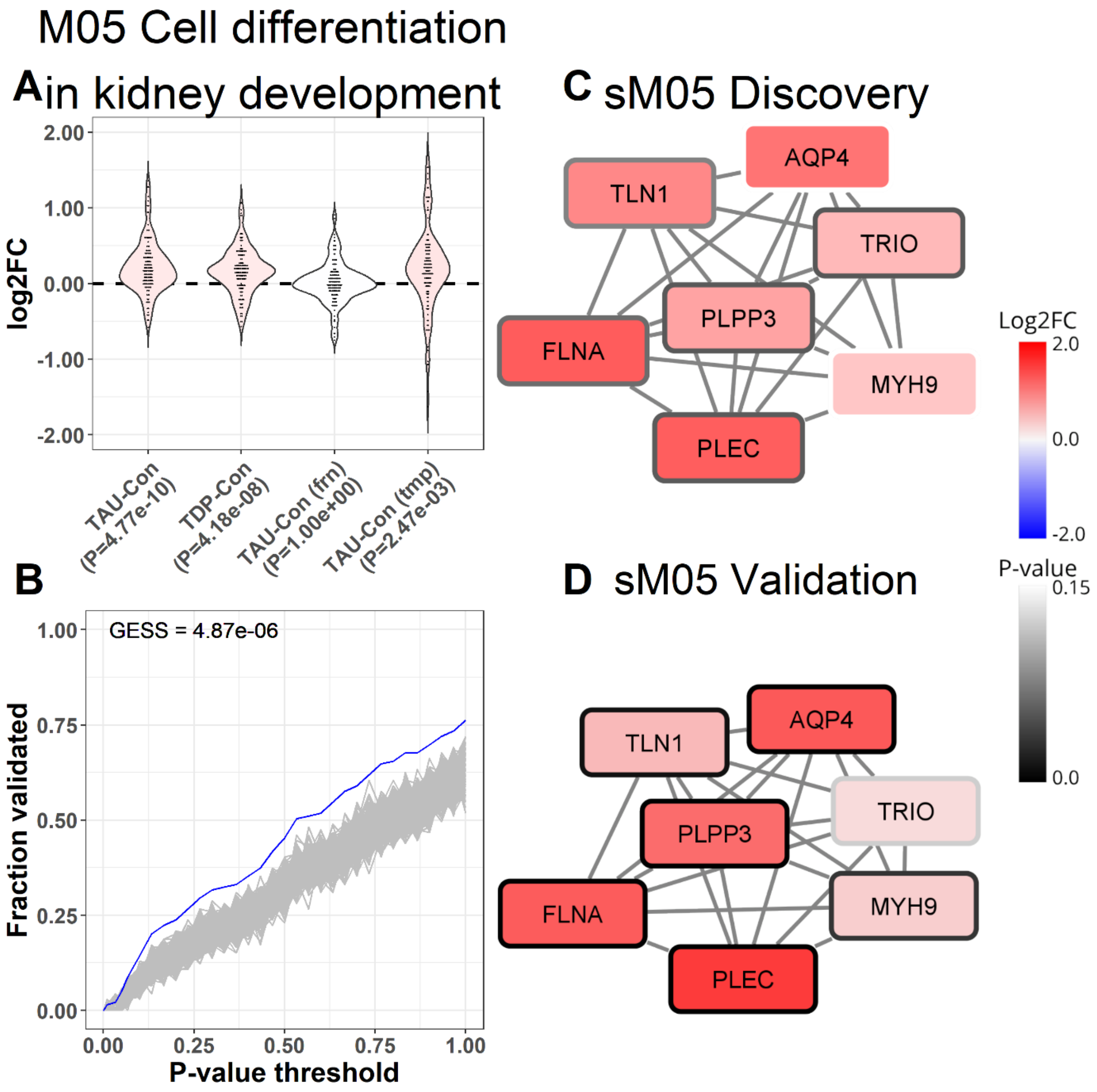
M05 Cell differentiation
A in kidney development
log2FC
2.00
1.00
0.00
-1.00
-2.00
TAU-Con (P=4.77e-10)
TDP-Con (P=4.18e-08)
TAU-Con (frn) (P=1.00e+00)
TAU-Con (tmp) (P=2.47e-03)
B
GESS = 4.87e-06
Fraction validated
1.00
0.75
0.50
0.25
0.00
P-value threshold
C sM05 Discovery
AQP4
TLN1
TRIO
PLPP3
FLNA
MYH9
PLEC
Log2FC
2.0
0.0
-2.0
D sM05 Validation
P-value
0.15
0.0
AQP4
TLN1
TRIO
PLPP3
FLNA
MYH9
PLEC

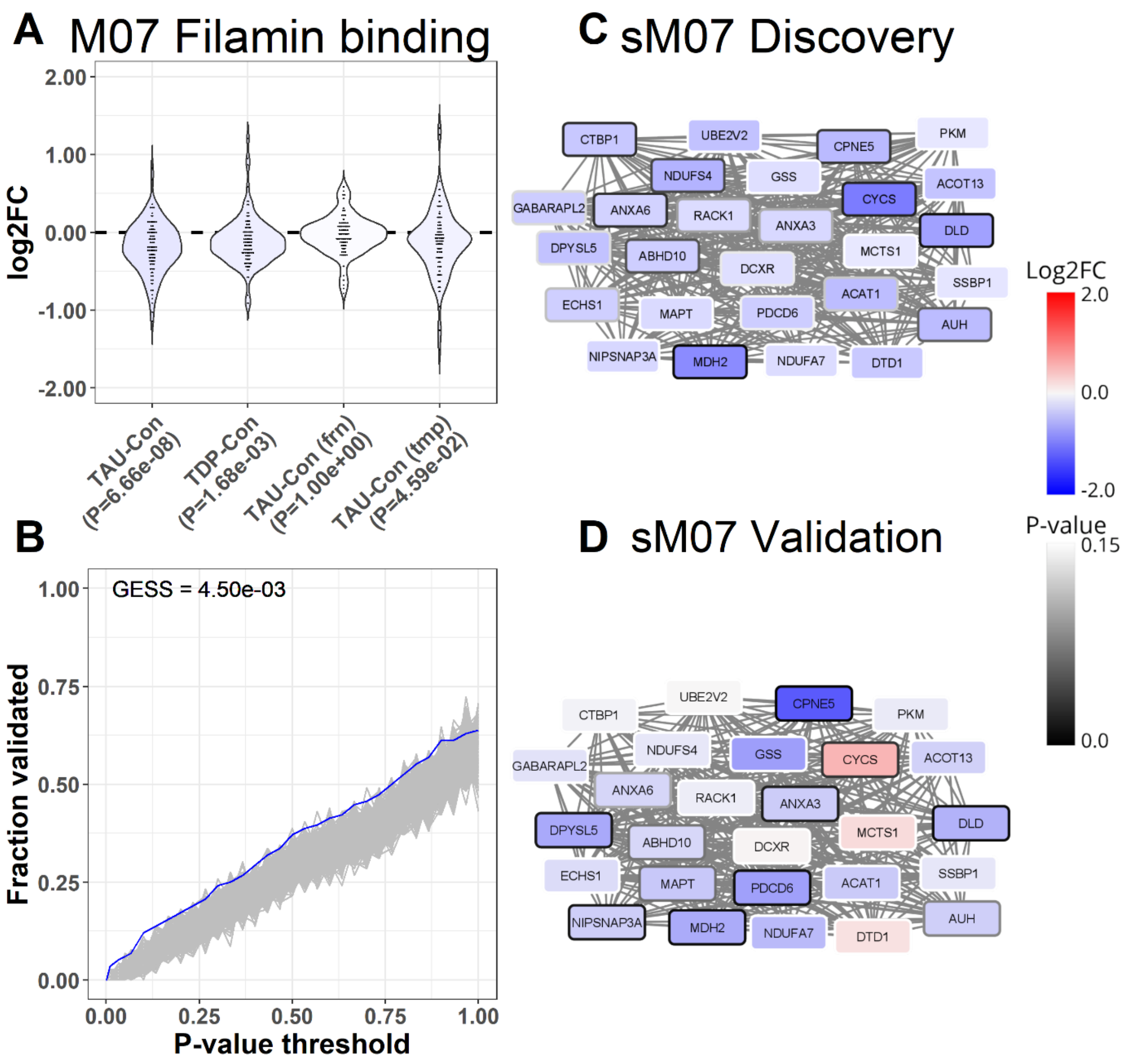
A M07 Filamin binding
log2FC
2.00
1.00
0.00
-1.00
-2.00
TAU-Con (P=6.66e-08)
TDP-Con (P=1.68e-03)
TAU-Con (frn) (P=1.00e+00)
TAU-Con (tmp) (P=4.59e-02)
B
GESS = 4.50e-03
Fraction validated
1.00
0.75
0.50
0.25
0.00
0.00
0.25
0.50
0.75
1.00
P-value threshold
C sM07 Discovery
D sM07 Validation
CTBP1
UBE2V2
CPNE5
PKM
NDUFS4
GSS
ACOT13
CYCS
GABARAPL2
ANXA6
RACK1
ANXA3
DLD
DPYSL5
ABHD10
DCXR
MCTS1
SSBP1
ECHS1
MAPT
PDCD6
ACAT1
AUH
NIPSNAP3A
MDH2
NDUFA7
DTD1
Log2FC
2.0
0.0
-2.0
P-value
0.15
0.0

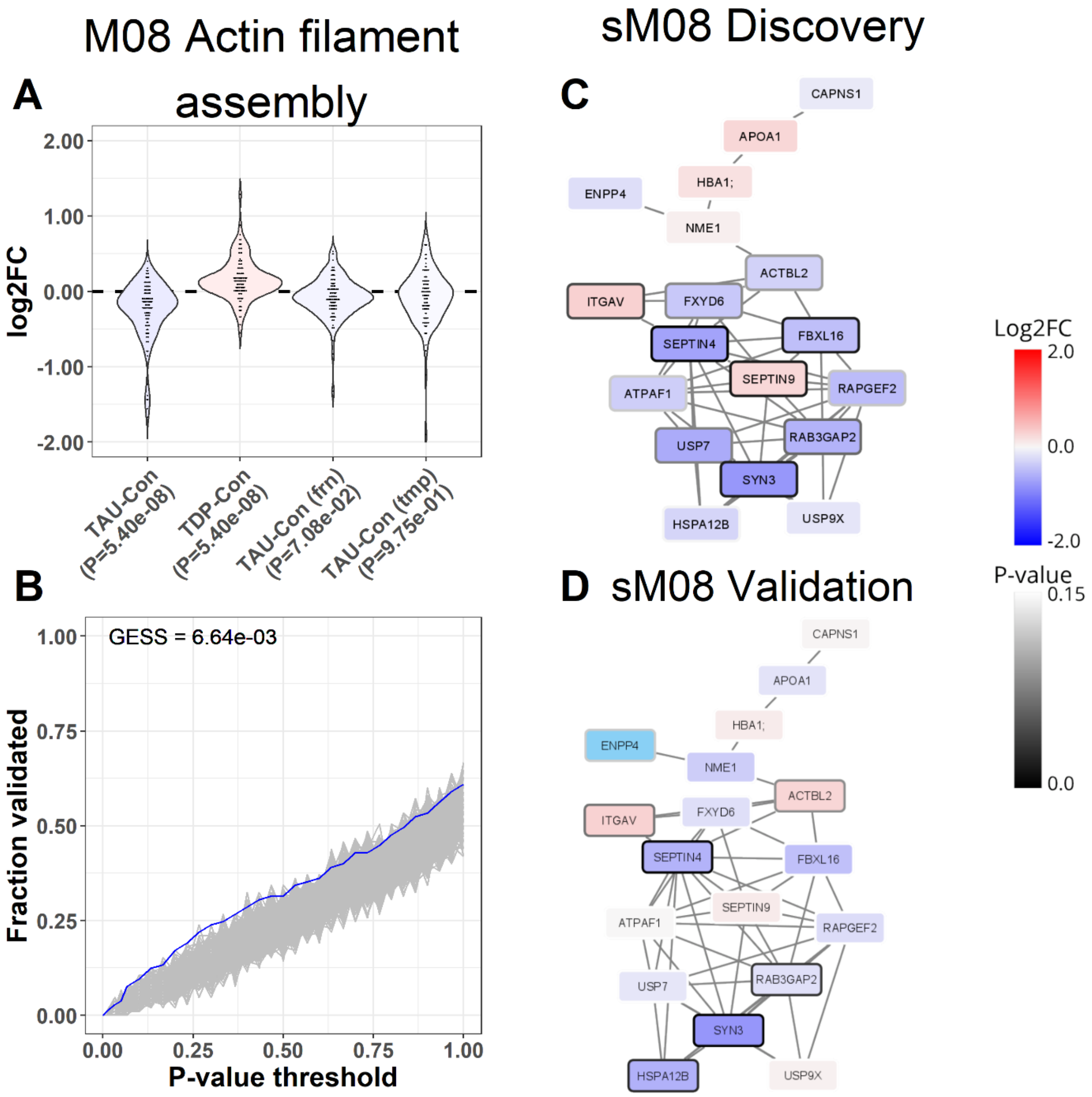
M08 Actin filament assembly
A
log2FC
2.00
1.00
0.00
-1.00
-2.00
TAU-Con (P=5.40e-08)
TDP-Con (P=5.40e-08)
TAU-Con (frn) (P=7.08e-02)
TAU-Con (tmp) (P=9.75e-01)
B
GESS = 6.64e-03
Fraction validated
1.00
0.75
0.50
0.25
0.00
0.00
0.25
0.50
0.75
1.00
P-value threshold
sM08 Discovery
C
CAPNS1
APOA1
HBA1;
ENPP4
NME1
ACTBL2
ITGAV
FXYD6
SEPTIN4
FBXL16
SEPTIN9
ATPAF1
RAPGEF2
RAB3GAP2
USP7
SYN3
HSPA12B
USP9X
D sM08 Validation
Log2FC
2.0
0.0
-2.0
P-value
0.15
0.0

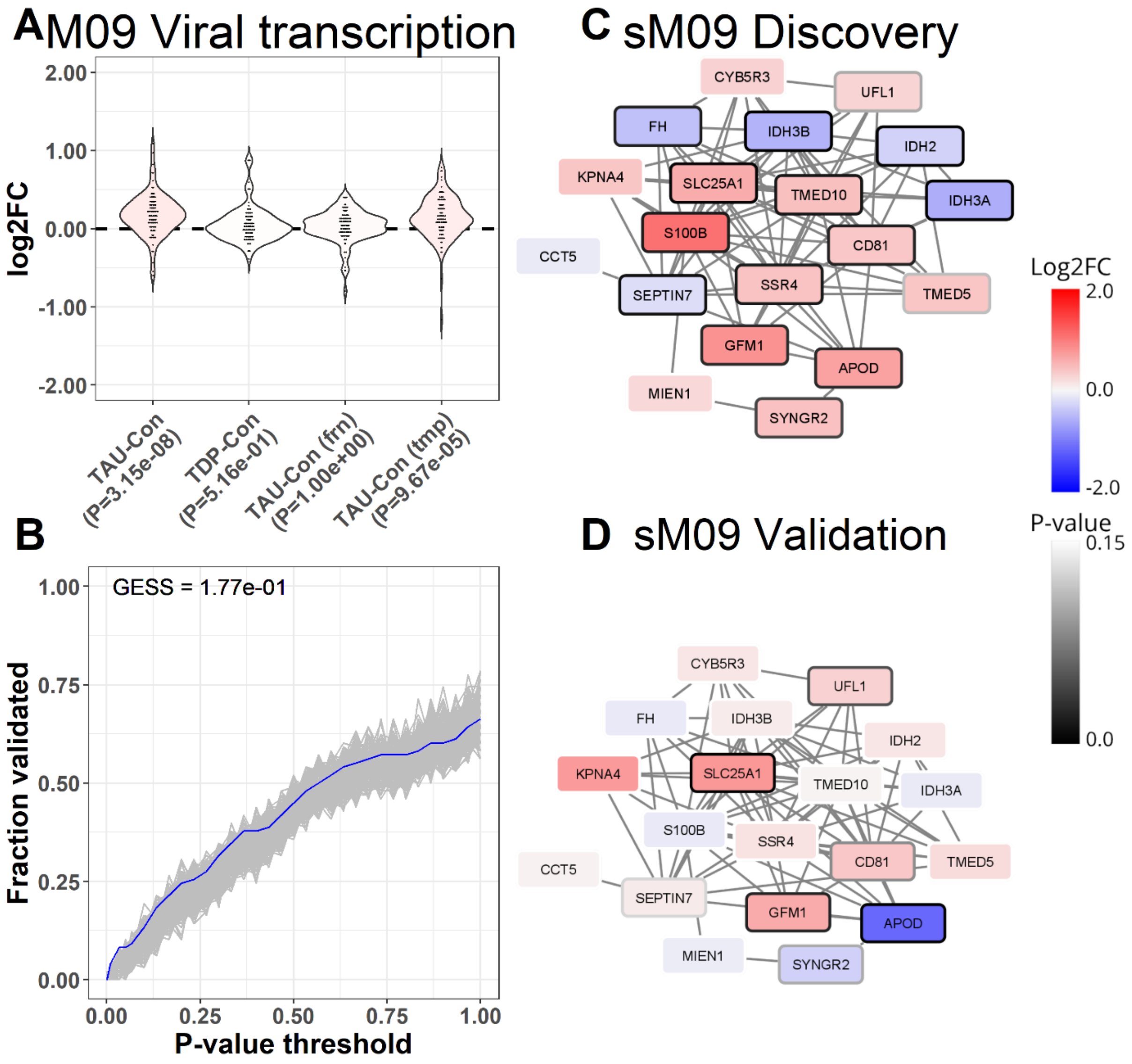

A M09 Viral transcription
log2FC
2.00
1.00
0.00
-1.00
-2.00
TAU-Con (P=3.15e-08)
TDP-Con (P=5.16e-01)
TAU-Con (frn) (P=1.00e+00)
TAU-Con (tmp) (P=9.67e-05)
B
GESS = 1.77e-01
Fraction validated
1.00
0.75
0.50
0.25
0.00
0.00
0.25
0.50
0.75
1.00
P-value threshold
C sM09 Discovery
CYB5R3
UFL1
FH
IDH3B
IDH2
KPNA4
SLC25A1
TMED10
IDH3A
S100B
CD81
CCT5
SEPTIN7
SSR4
TMED5
GFM1
APOD
MIEN1
SYNGR2
Log2FC
2.0
0.0
-2.0
P-value
0.15
0.0
D sM09 Validation
CYB5R3
UFL1
FH
IDH3B
IDH2
KPNA4
SLC25A1
TMED10
IDH3A
S100B
SSR4
CD81
TMED5
CCT5
SEPTIN7
GFM1
APOD
MIEN1
SYNGR2

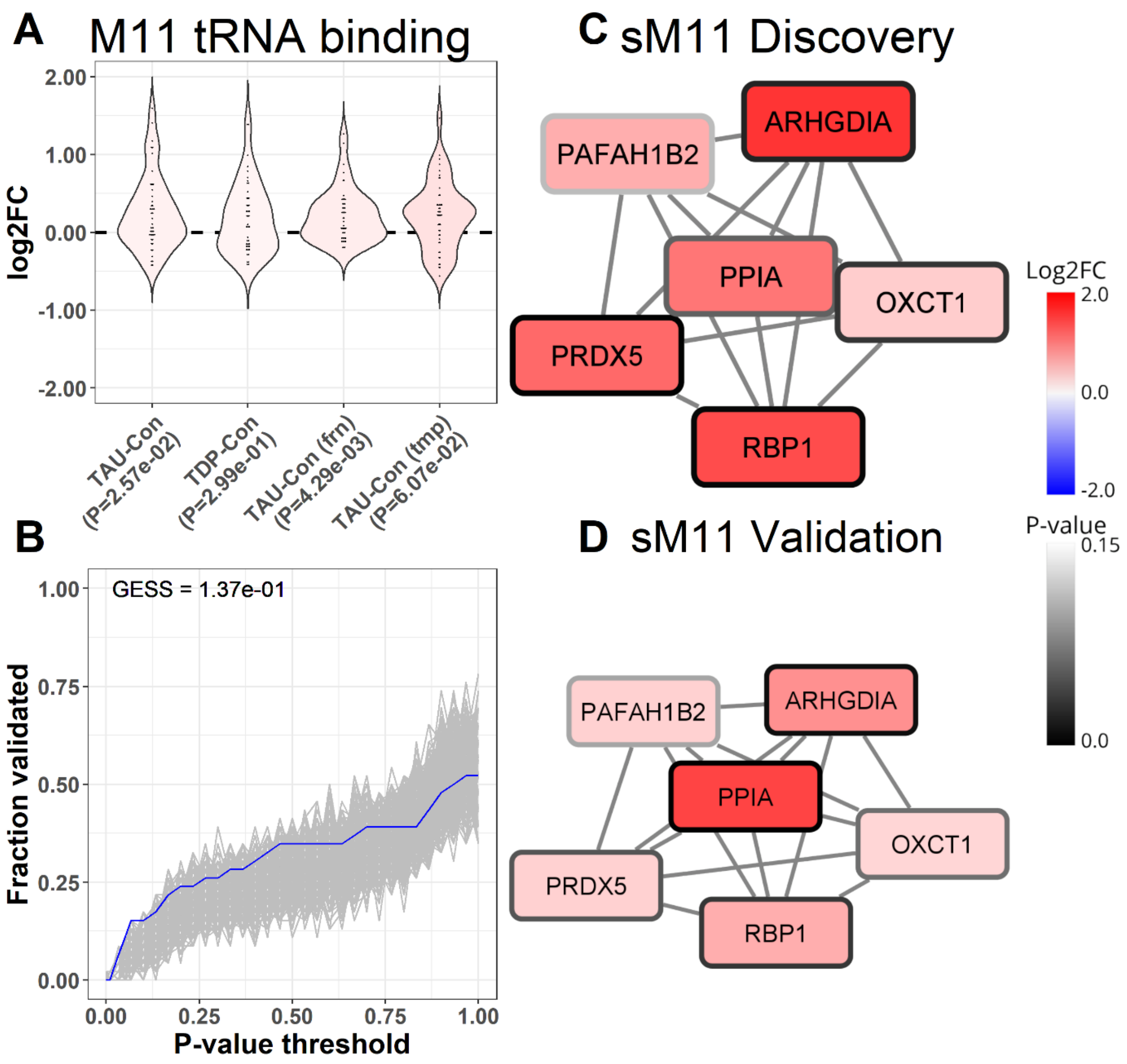
A
M11 tRNA binding
log2FC
2.00
1.00
0.00
-1.00
-2.00
TAU-Con (P=2.57e-02)
TDP-Con (P=2.99e-01)
TAU-Con (frn) (P=4.29e-03)
TAU-Con (tmp) (P=6.07e-02)
B
GESS = 1.37e-01
Fraction validated
1.00
0.75
0.50
0.25
0.00
0.00
0.25
0.50
0.75
1.00
P-value threshold
C
sM11 Discovery
ARHGDIA
PAFAH1B2
PPIA
OXCT1
PRDX5
RBP1
Log2FC
2.0
0.0
-2.0
D
sM11 Validation
P-value
0.15
0.0
PAFAH1B2
ARHGDIA
PPIA
OXCT1
PRDX5
RBP1

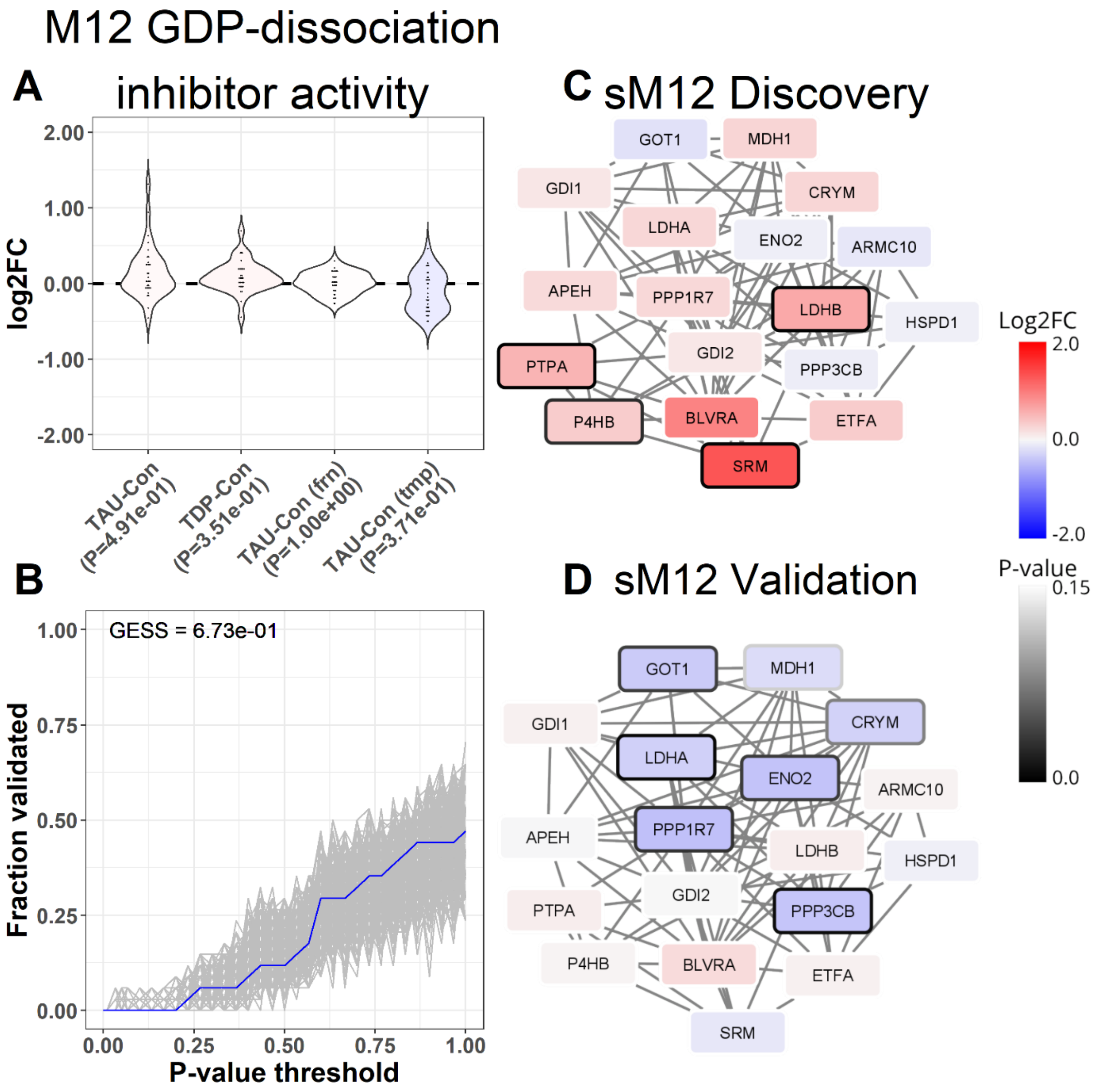
M12 GDP-dissociation inhibitor activity
A
log2FC
2.00
1.00
0.00
-1.00
-2.00
TAU-Con (P=4.91e-01)
TDP-Con (P=3.51e-01)
TAU-Con (frn) (P=1.00e+00)
TAU-Con (tmp) (P=3.71e-01)
B
GESS = 6.73e-01
Fraction validated
1.00
0.75
0.50
0.25
0.00
P-value threshold
C sM12 Discovery
GOT1
MDH1
GDI1
CRYM
LDHA
ENO2
ARMC10
APEH
PPP1R7
LDHB
HSPD1
PTPA
GDI2
PPP3CB
P4HB
BLVRA
ETFA
SRM
Log2FC
2.0
0.0
-2.0
P-value
0.15
0.0
D sM12 Validation

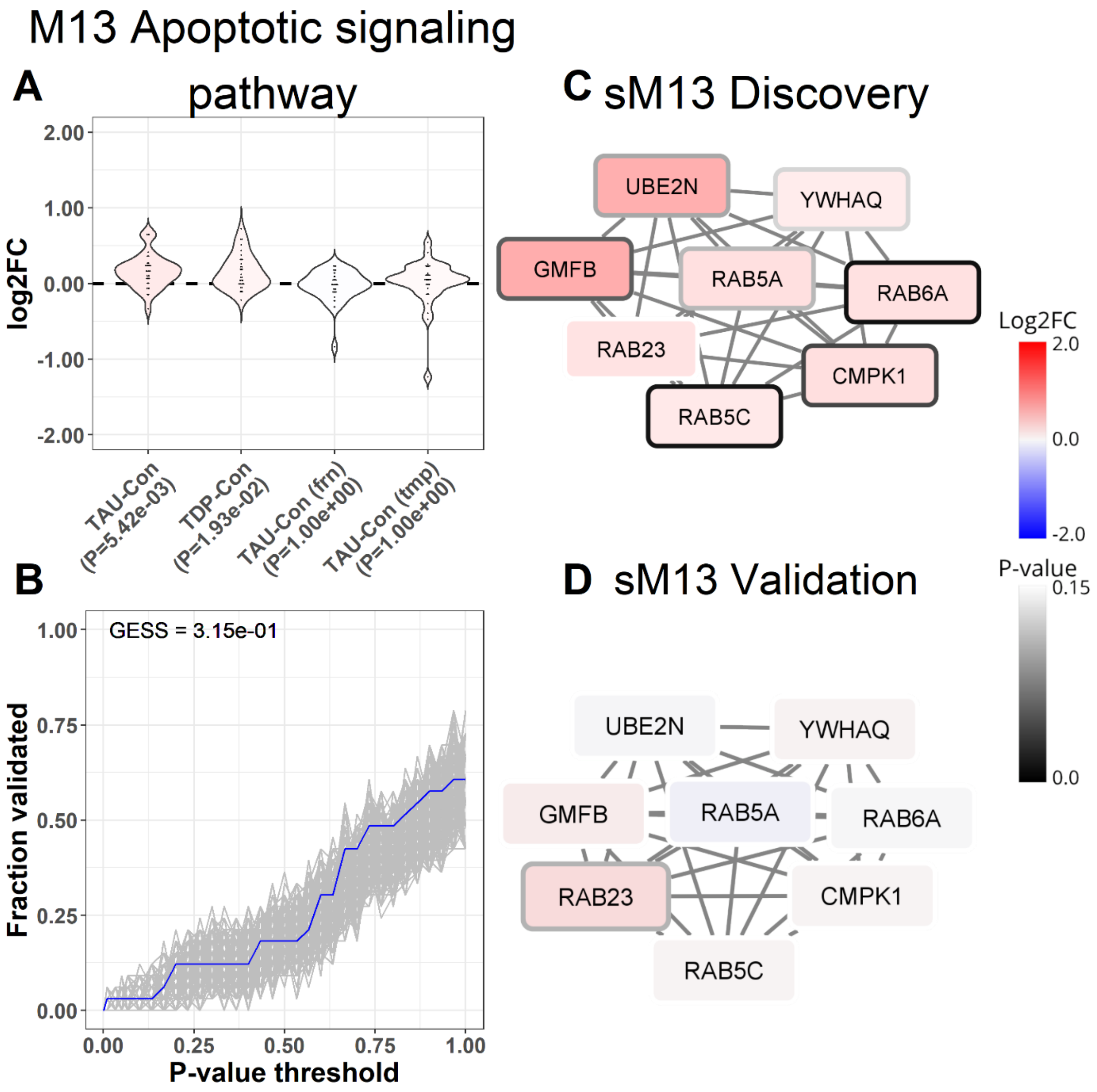

M13 Apoptotic signaling pathway
A
log2FC
2.00
1.00
0.00
-1.00
-2.00
TAU-Con (P=5.42e-03)
TDP-Con (P=1.93e-02)
TAU-Con (frn) (P=1.00e+00)
TAU-Con (tmp) (P=1.00e+00)
B
GESS = 3.15e-01
Fraction validated
1.00
0.75
0.50
0.25
0.00
0.00
0.25
0.50
0.75
1.00
P-value threshold
C sM13 Discovery
UBE2N
YWHAQ
GMFB
RAB5A
RAB6A
RAB23
CMPK1
RAB5C
Log2FC
2.0
0.0
-2.0
D sM13 Validation
UBE2N
YWHAQ
GMFB
RAB5A
RAB6A
RAB23
CMPK1
RAB5C
P-value
0.15
0.0

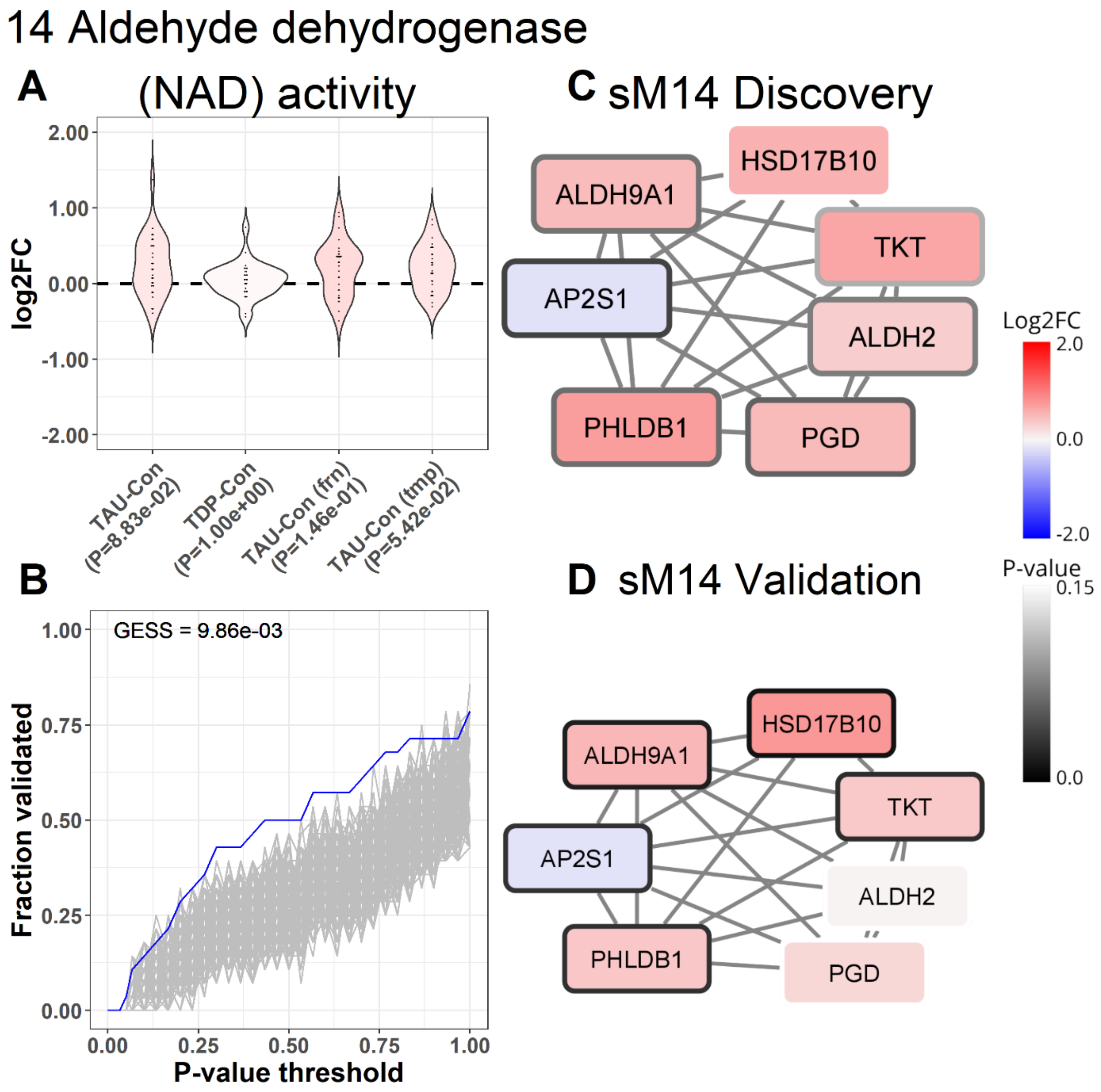
14 Aldehyde dehydrogenase
(NAD) activity
A
log2FC
2.00
1.00
0.00
-1.00
-2.00
TAU-Con (P=8.83e-02)
TDP-Con (P=1.00e+00)
TAU-Con (frn) (P=1.46e-01)
TAU-Con (tmp) (P=5.42e-02)
B
GESS = 9.86e-03
Fraction validated
1.00
0.75
0.50
0.25
0.00
0.00
0.25
0.50
0.75
1.00
P-value threshold
C sM14 Discovery
HSD17B10
ALDH9A1
TKT
AP2S1
ALDH2
PHLDB1
PGD
Log2FC
2.0
0.0
-2.0
P-value
0.15
0.0
D sM14 Validation
HSD17B10
ALDH9A1
TKT
AP2S1
ALDH2
PHLDB1
PGD

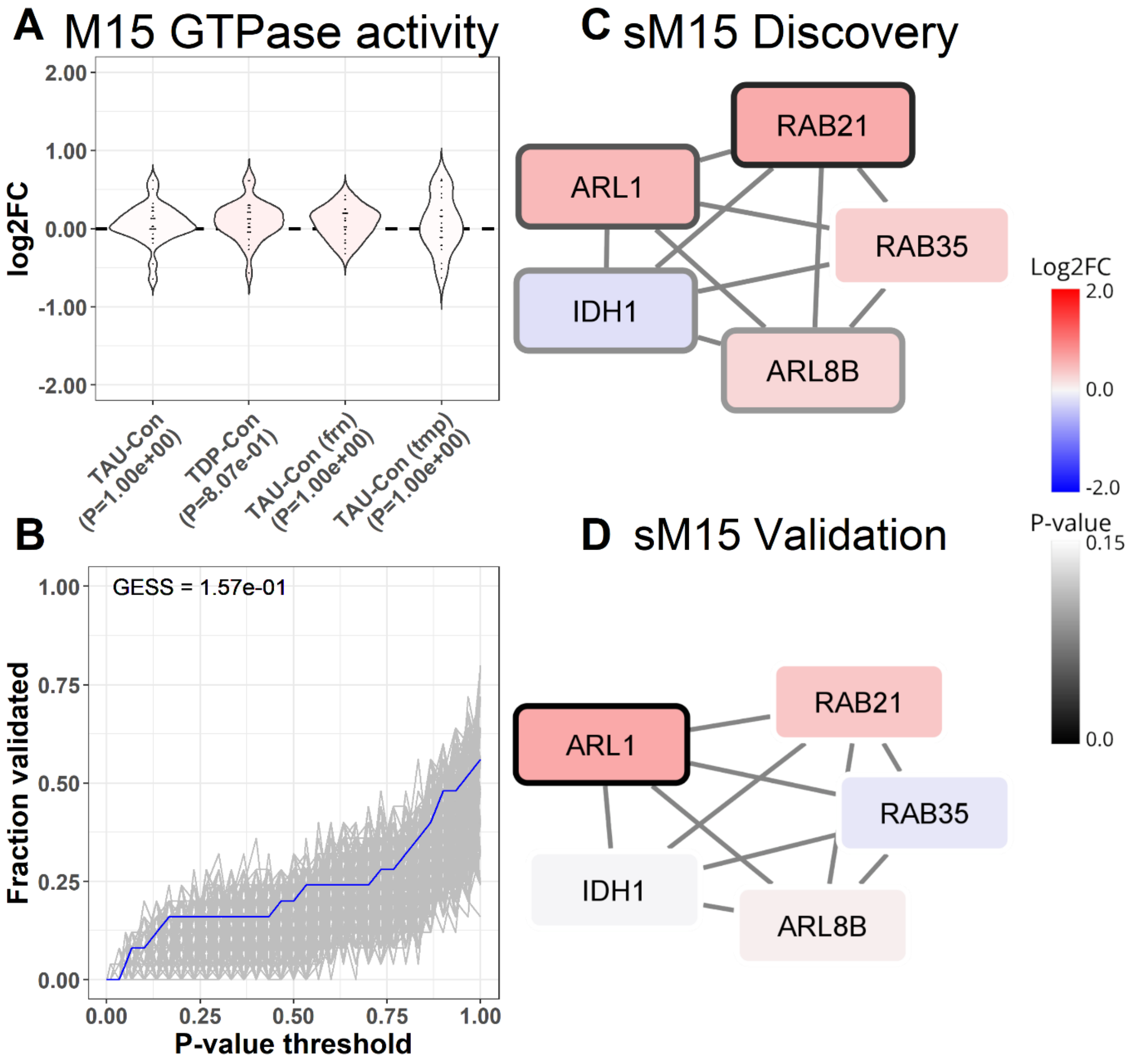
A M15 GTPase activity
log2FC
2.00
1.00
0.00
-1.00
-2.00
TAU-Con (P=1.00e+00)
TDP-Con (P=8.07e-01)
TAU-Con (frn) (P=1.00e+00)
TAU-Con (tmp) (P=1.00e+00)
B
GESS = 1.57e-01
Fraction validated
1.00
0.75
0.50
0.25
0.00
0.00
0.25
0.50
0.75
1.00
P-value threshold
C sM15 Discovery
RAB21
ARL1
RAB35
IDH1
ARL8B
Log2FC
2.0
0.0
-2.0
P-value
0.15
0.0
D sM15 Validation
RAB21
ARL1
RAB35
IDH1
ARL8B

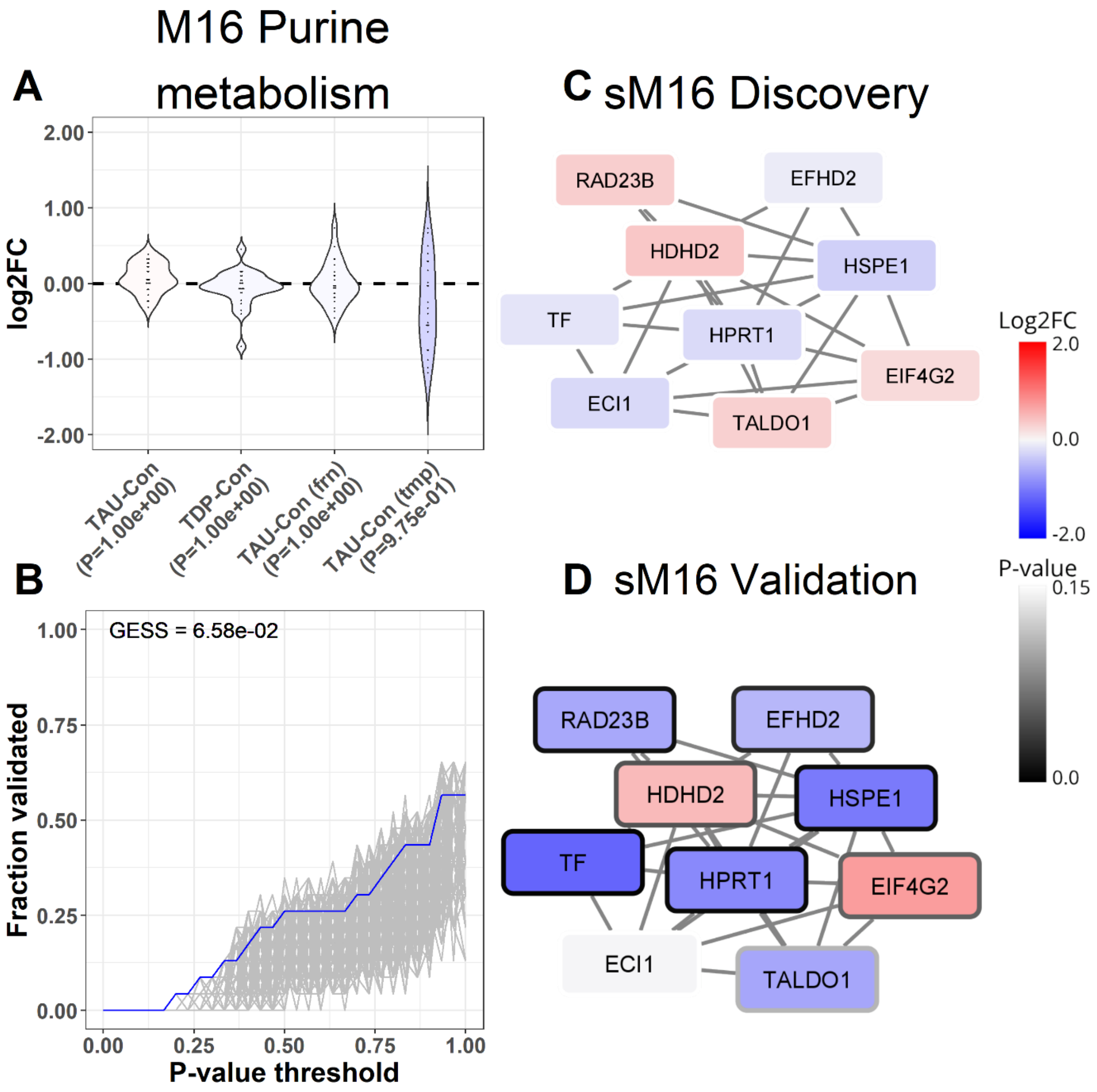
A
M16 Purine metabolism
log2FC
2.00
1.00
0.00
-1.00
-2.00
TAU-Con (P=1.00e+00)
TDP-Con (P=1.00e+00)
TAU-Con (frn) (P=1.00e+00)
TAU-Con (tmp) (P=9.75e-01)
B
GESS = 6.58e-02
Fraction validated
1.00
0.75
0.50
0.25
0.00
0.00
0.25
0.50
0.75
1.00
P-value threshold
C sM16 Discovery
RAD23B
EFHD2
HDHD2
HSPE1
TF
HPRT1
EIF4G2
ECI1
TALDO1
Log2FC
2.0
0.0
-2.0
P-value
0.15
0.0
D sM16 Validation
RAD23B
EFHD2
HDHD2
HSPE1
TF
HPRT1
EIF4G2
ECI1
TALDO1

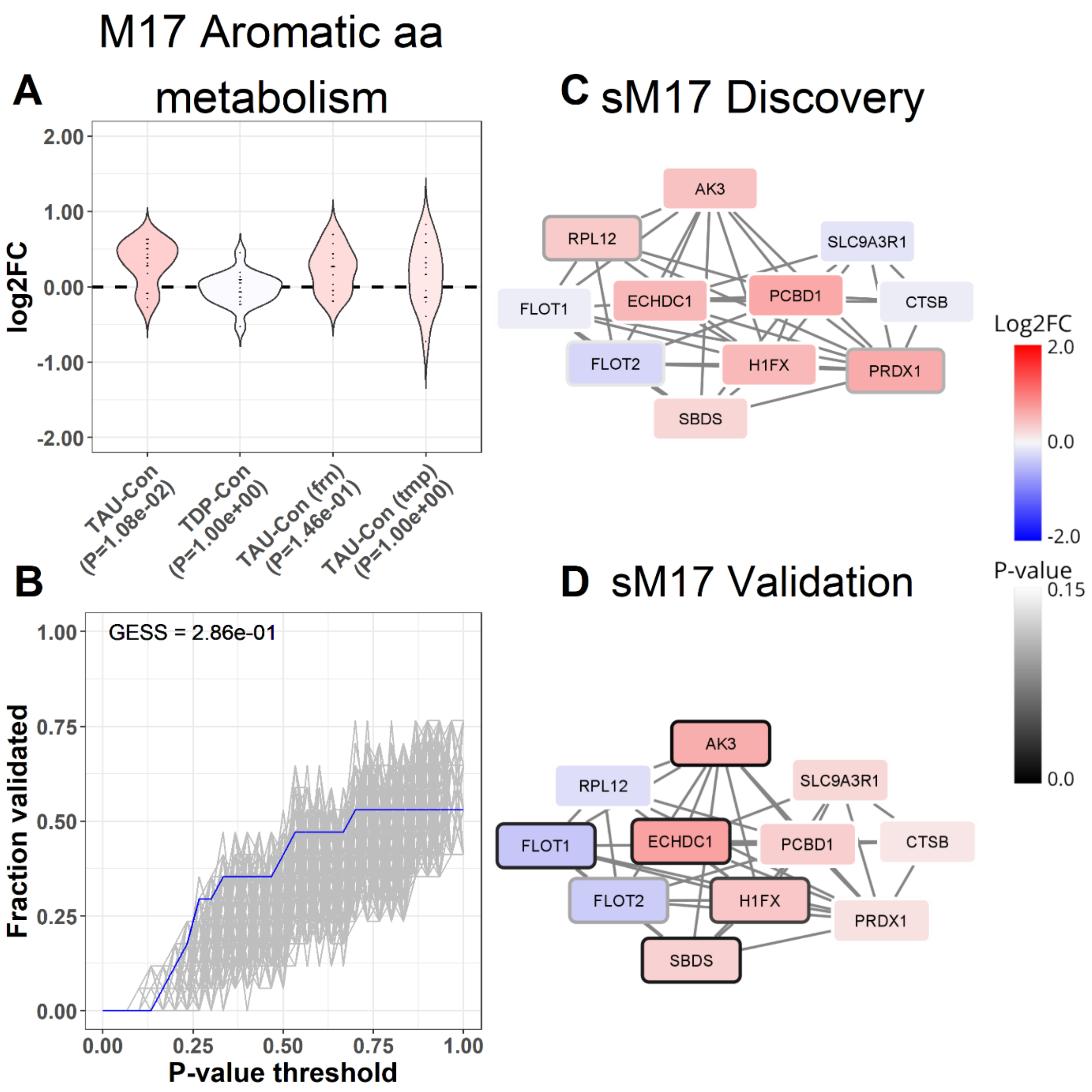

M17 Aromatic aa metabolism
A
log2FC
2.00
1.00
0.00
-1.00
-2.00
TAU-Con (P=1.08e-02)
TDP-Con (P=1.00e+00)
TAU-Con (frn) (P=1.46e-01)
TAU-Con (tmp) (P=1.00e+00)
B
GESS = 2.86e-01
Fraction validated
1.00
0.75
0.50
0.25
0.00
P-value threshold
C sM17 Discovery
AK3
RPL12
SLC9A3R1
FLOT1
ECHDC1
PCBD1
CTSB
FLOT2
H1FX
PRDX1
SBDS
Log2FC
2.0
0.0
-2.0
P-value
0.15
0.0
D sM17 Validation

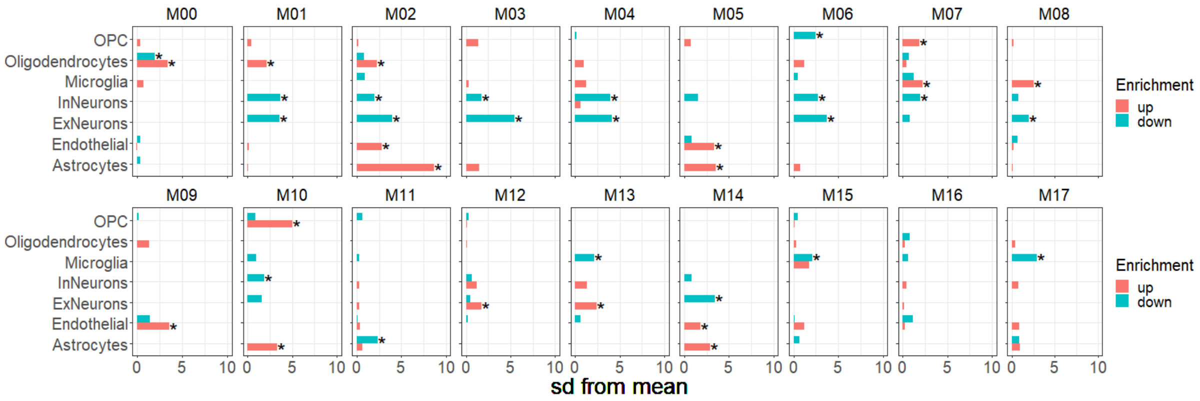

Figure S17 Cell type enrichment in all modules. Cell type enrichment in each of the modules was determined based on the log2FC values of specific CNS cell type protein markers. Included cell types are astrocytes, endothelial cells, excitatory neurons, inhibitory neurons, microglia, oligodendrocytes and oligodendrocyte precursor cells. Enrichment up (red bar) means that the markers for that cell type had high log2FC values in the FTLD-tau samples compared to the control group, enrichment down (blue bar) means that the markers for that cell type had low log2FC values in the FTLD-tau samples compared to the control group.

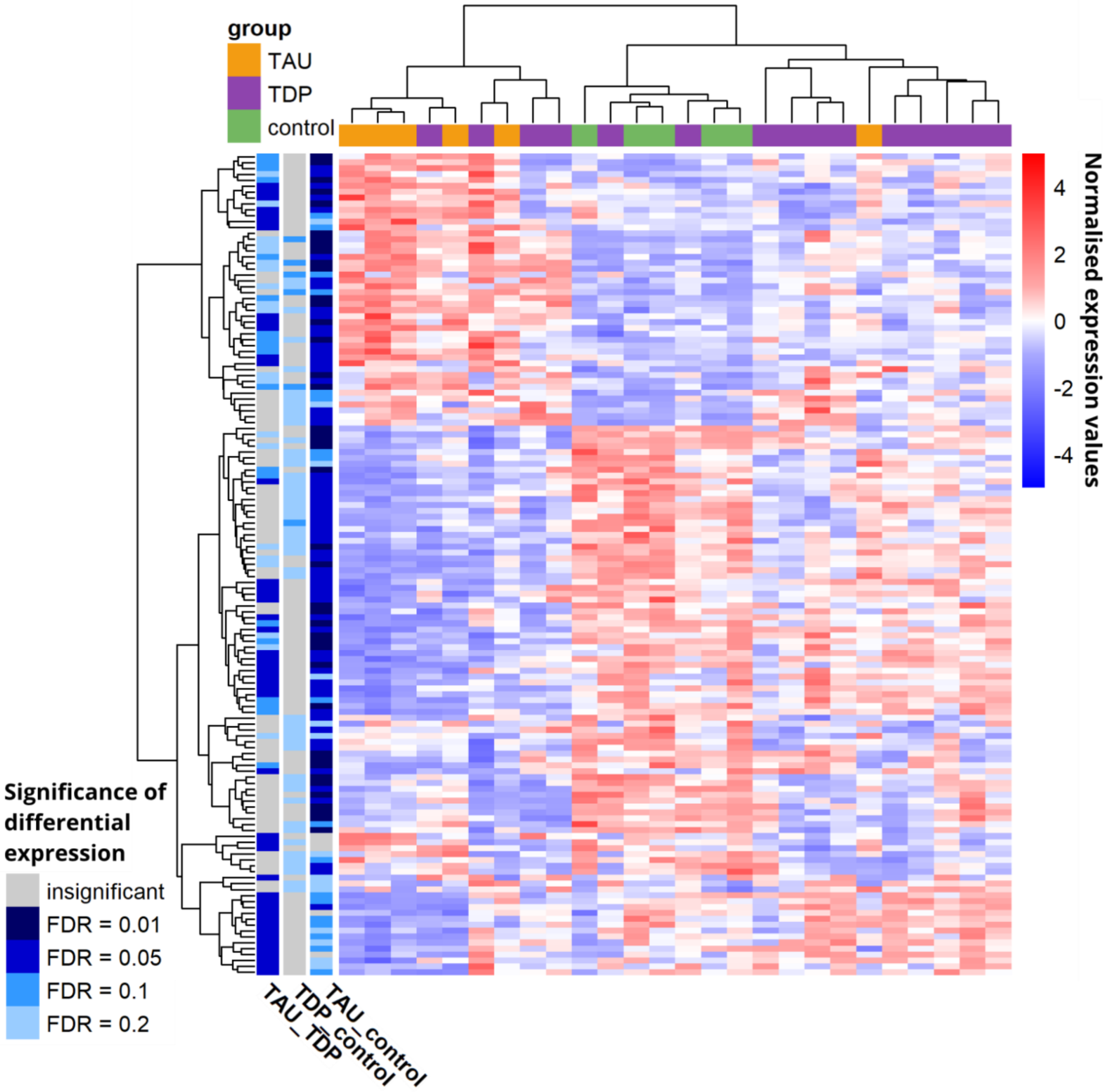

Figure S18 Hierarchical clustering of the samples of the discovery cohort based on the abundance levels of the 50 most significantly differentially expressed proteins resulting from the 3 pairwise comparisons using Spearman correlation. The samples are represented on the x-axis, FTLD-tau samples in yellow, FTLD-TDP in purple, and NHC in green. Normalised protein abundance levels are represented on the y-axis. Shades of red represent upregulated proteins, and shades of blue downregulated proteins. P-values for each 3 pairwise comparisons are indicated to the left of the figure. Shades of blue indicate statistically significant values; the darker the more significant, and gray indicates non-significant p-values.

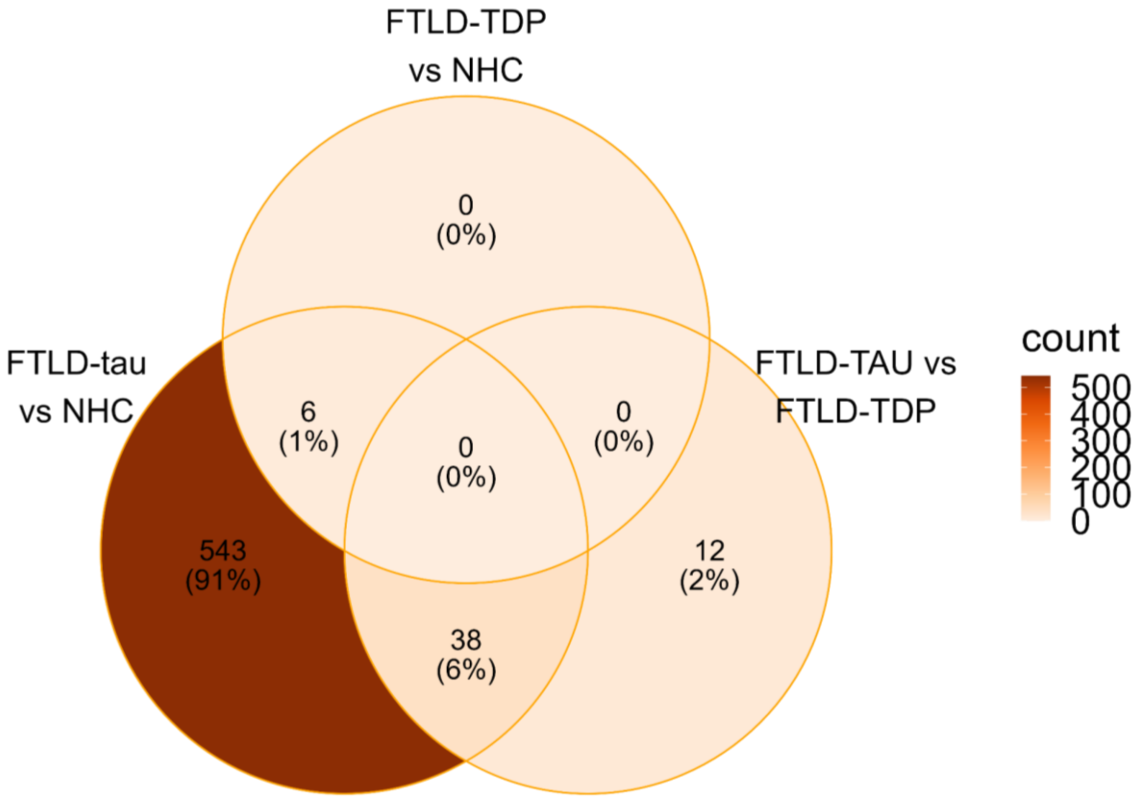

Figure S19 Overlap in significant proteins between the different pairwise comparisons. A significance cutoff at FDR=0.1 was used here. The figure shows that most proteins significantly different in FTLD-TDP vs NHC and FTLD-tau vs FTLD-TDP are also significant in FTLD-tau vs NHC. There are 12 proteins significantly different between FTLD-tau and FTLD-TDP that are not captured in FTLD- tau vs NHC.

Table S1 Discovery and validation cohorts. mfg, medial frontal gyrus; temp, temporal lobe; NHC, neurologically healthy control; AD, autosomal dominant; S, sporadic; Fa, familial; NA, not applicable; f, female; m, male; PMI, postmortem interval in hours and minutes. Age is in years.

Cohort	Anatomical regions	PathologY (TDP subtype)	genotype (mutation)	Age	sex	PMI
Discovery (n=26)	mfg	Tau	AD (MAPT)	68	m	05:25
	mfg	Tau	AD (MAPT)	54	F	05:40
	mfg	Tau	AD (MAPT)	64	F	05:10
	mfg	Tau	AD (MAPT)	46	M	05:35
	mfg	Tau	AD (MAPT)	61	F	06:00
	mfg	Tau	AD (MAPT)	56	m	04:05
	mfg	TDP43 (B)	AD (C9ORF)	63	m	04:45
	mfg	TDP43 (ND)	AD (C9ORF)	58	m	17:30
	mfg	TDP43 (C)	AD (C9ORF)	64	m	06:20
	mfg	TDP43 (B)	AD (C9ORF)	52	f	08:15
	mfg	TDP43 (ND)	AD (C9ORF)	60	f	06:20
	mfg	TDP43 (A)	AD (C9ORF)	70	f	04:40
	mfg	TDP43 (A)	AD (C9ORF)	75	f	05:15
	mfg	TDP43 (B)	AD (C9ORF)	75	m	06:25
	mfg	TDP43 (A)	AD (GRN)	69	f	06:50
	mfg	TDP43 (A)	AD (GRN)	59	m	04:45
	mfg	TDP43 (A)	S	57	f	07:20
	mfg	TDP43 (C)	S	68	f	06:35
	mfg	TDP43 (C)	S	69	M	06:45
	mfg	TDP43 (C)	Fa (none identified)	67	M	05:45
	mfg	TDP43 (C)	Fa (none identified)	69	m	05.20
	mfg	NHC	NA	82	M	12:55
	mfg	NHC	NA	75	F	07:45
	mfg	NHC	NA	55	M	07:15
	mfg	NHC	NA	57	F	07:40
	mfg	NHC	NA	60	f	08:10
Validation (N=24)	mfg and temp	Tau	AD (MAPT)	66	M	05:00
	mfg and temp	Tau	AD (MAPT)	51	M	04:25
	mfg and temp	Tau	AD (MAPT)	60	M	05:23
	mfg and temp	Tau	AD (MAPT)	64	F	07:30
	mfg and temp	Tau	AD (MAPT)	60	M	04:40
	mfg and temp	Tau	AD (MAPT)	67	F	04:10
	mfg and temp	Tau	AD (MAPT)	75	F	07:25
	mfg and temp	Tau	AD (MAPT)	66	F	06:40
	mfg and temp	Tau	AD (MAPT)	54	F	07:30
	mfg and temp	Tau	AD (MAPT)	49	M	05:10
	mfg and temp	Tau	AD (MAPT)	52	M	11:30
	temp	Tau	AD (MAPT)	64	M	06:25
	temp	Tau	AD (MAPT)	46	M	05:35
	mfg and temp	NHC	NA	91	M	08:00
	mfg and temp	NHC	NA	84	F	05:36
	mfg and temp	NHC	NA	83	F	06:03
	mfg and temp	NHC	NA	60	F	07:30
	mfg and temp	NHC	NA	70	M	03:35
	mfg and temp	NHC	NA	83	M	05:45
	mfg and temp	NHC	NA	76	F	07:15
	mfg and temp	NHC	NA	89	F	05:40
	mfg	NHC	NA	82	M	05:50
	mfg	NHC	NA	87	F	07:00
	mfg	NHC	NA	75	F	05:25

Table S2 Module summary. Module identity (number), module size and largest submodule size (number of proteins in the module/submodule), and validation in the FTLD-tau medial frontal gyrus cortex or temporal cortex data set of the validation cohort. Module function indicates the Gene ontology (GO) term enrichment analysis biological function most significantly associated with the module. The last column indicates the number of proteins in the subnetwork that are associated with the GO term listed in the column "Module function".

#	Module size	Subnetwork size	Module validated in the frontal cortex validation data set	Module validated in the temporal cortex validation data set	Module function	Number of proteins in subnetwork associated with module function
M00	26	5	No	No	--- (not a module)	-
M01	272	12	Yes	Yes	Transmembrane transporter activity	0
M02	233	12	Yes	Yes	Chromatin regulation	0
M03	212	13	Yes	Yes	Postsynapse organisation	1
M04	159	26	Yes	Yes	Oxidative phosphorylation	5
M05	148	7	No	Yes	Protein-containing complex binding	0
M06	129	37	Yes	Yes	Regulation of vesicle-mediated transport	8
M07	124	27	No	Yes	Filamin binding	0
M08	121	18	No	No	Positive regulation of actin filament bundle assembly	1
M09	103	19	No	Yes	Co-translational protein targeting to membrane	0
M10	56	25	Yes	Yes	Phenylalanine-tRNA ligase activity	1
M11	49	6	Yes	No	tRNA binding	0
M12	34	18	No	No	Carbohydrate metabolic process	2
M13	34	8	No	No	Apoptotic signalling pathway	1
M14	30	7	Yes	Yes	Aldehyde dehydrogenase (NAD) activity	2
M15	28	5	No	No	GTPase activity	3
M16	25	9	No	No	Superoxide metabolic process	1
M17	18	11	No	No	Oxidoreductase activity	1

Table S3 Mean age, PMI and gender distribution in the three pathological groups. No significant differences in these variables were found between the three groups.

	Mean age	f/m	%f	mean PMI (h)
NHC	65	3/2	0.60	8.8
FTLD-TDP	65	7/8	0.47	6.9
FTLD-tau	58	3/3	0.50	5.3
